# Lifestyle and Dietary Behaviors Are Associated with Body Mass Index in Romanian Young Adults

**DOI:** 10.3390/nu18101644

**Published:** 2026-05-21

**Authors:** Diana Crișan, Oleg Frumuzachi, Denisia Pașca, Laura Gavrilaș, Gianina Crișan

**Affiliations:** 1 Department of Pharmaceutical Botany, Faculty of Pharmacy, “Iuliu Hațieganu” University of Medicine and Pharmacy, 23 Gheorghe Marinescu Street, 400337 Cluj-Napoca, Romania; diana.suciu.crisan@elearn.umfcluj.ro (D.C.); gcrisan@umfcluj.ro (G.C.); 2 The Romanian Dietitians Association, 6 Dionisie Roman Street, 400595 Cluj-Napoca, Romania; denisia.pasca@umfcluj.ro; 3 Department 2, Faculty of Nursing and Health Sciences, “Iuliu Hațieganu” University of Medicine and Pharmacy, 23 Gheorghe Marinescu Street, 400337 Cluj-Napoca, Romania

**Keywords:** meal regularity, obesogenic behaviors, soft-drink intake, beverage consumption, nationwide cross-sectional survey

## Abstract

**Background/Objectives:** Overweight and obesity are increasing globally. However, structured contemporary data on lifestyle behaviors and adiposity in Romanian young adults remain limited. Therefore, this study aimed to describe dietary and lifestyle habits, BMI, and overweight/obesity prevalence in Romanian adults aged 18–30 years and to examine associations between these variables. **Methods:** This cross-sectional online questionnaire study included 1202 young Romanian adults. BMI was calculated from self-reported height and weight and analyzed continuously, as well as for overweight/obesity (BMI ≥ 25 kg/m^2^). Pre-specified exposures were compulsive eating, soft-drink intake, breakfast frequency, physical activity, and sleep duration. Multivariable linear regression with heteroscedasticity-consistent standard errors was used for BMI, and modified Poisson regression with robust variance was used for overweight/obesity. Composite dietary score, sex-interaction, and sensitivity analyses were also performed. **Results:** Mean age was 23.2 ± 3.3 years, mean BMI was 23.8 ± 4.2 kg/m^2^, and 32.4% of participants had overweight/obesity. Men had higher BMI and a higher prevalence of overweight/obesity than women. Compulsive eating and soft-drink intake showed dose-dependent associations with higher BMI and higher overweight/obesity prevalence. Short sleep duration (≤5 h/night) and daily breakfast consumption were associated with a higher and, respectively, lower prevalence of overweight/obesity. Physical activity showed no independent association after full adjustment, although this finding may be influenced by the use of a single self-reported item. Composite-score analyses supported the main findings. **Conclusions:** In Romanian young adults, compulsive eating and soft-drink intake were the most consistent behavioral correlates of adiposity, while breakfast regularity and short sleep showed threshold-type associations with overweight/obesity. These findings may inform the design of multicomponent prevention strategies, although longitudinal confirmation is needed.

## 1. Introduction

Overweight and obesity have become one of the defining public-health challenges of the twenty-first century. Between 1990 and 2022, adult obesity prevalence more than doubled among women and nearly tripled among men worldwide, and more than one billion people now live with obesity [1,2]. The WHO European Region is particularly affected: almost 60% of adults are overweight or living with obesity, and no Member State was on track to halt the rise in obesity by 2025 [3]. These trends translate into increased risks of type 2 diabetes, cardiovascular disease, several cancers, and premature mortality, highlighting the need to characterize modifiable behavioral risk factors at the population level [4,5,6,7].

Young adulthood (18–30 years) has been repeatedly described as an important period for disease prevention, being a critical window yet an overlooked one for weight-related behavioral changes [8,9]. The transition from adolescence to independent adulthood is typically accompanied by a rapid reorganization of lifestyle: more autonomous food choices, irregular meal patterns, increased sedentary study and work, frequent alcohol and soft-drink consumption, and exposure to academic and occupational stressors [10,11]. Behaviors and weight trajectories established during this period tend to perpetuate into later adulthood and shape long-term cardiometabolic risk [12], making young adults an important target group for public health programs.

Several dietary and lifestyle exposures have been consistently linked to higher BMI in this age group. Carbonated beverage/soft-drink intake showed a dose–response association with weight gain in adults and is among the most policy-relevant modifiable risk factors for obesity prevention [13,14]. Disinhibited forms of intake, in particular emotional and compulsive eating, are associated with greater consumption of energy-dense, hyperpalatable foods and with higher BMI, and adults living with obesity report emotional eating more frequently than adults of healthy weight [15,16]. Breakfast skipping has been associated with a modestly elevated risk of overweight and obesity in observational studies across diverse settings [17], while low physical activity and short sleep duration have been correlated with the incidence of metabolic syndrome and type 2 diabetes [18,19,20]. The interplay between sleep patterns and dietary behaviors may further influence somatic health indicators, including body composition and cardiovascular risk markers in young adults [21]. Importantly, BMI alone may not fully capture adiposity-related health risks, as individuals with normal BMI may still present excess body fat and adverse metabolic profiles [22].

In Eastern Europe, as well as in the rest of Europe, the prevalence of overweight and obesity has markedly increased over time, with Romania recording one of the largest increases in adult obesity prevalence among men worldwide (+31.7 percentage points from 1990 to 2022) [1]. The national PREDATORR study documented an age- and sex-adjusted obesity prevalence of 31.9% and abdominal obesity of 73.9% in adults aged 20–79 years, together with a high prevalence of metabolic syndrome [23]. A cross-sectional survey reported that obesity had a prevalence of 9.9% in the 18–39-year age group, with “irregular meals” and “eating while watching television” being among the most frequent unhealthy habits [24]. Data in younger Romanians have largely focused on school-aged populations: a survey of Transylvanian children and adolescents (5–17 years) identified that 13.6% and 22.2% had overweight and obesity [25], respectively, while another survey including adolescents (average age 15.5 years) identified poor diet quality, high screen time, and low physical activity as key correlates of unfavorable nutritional status [26].

Nonetheless, to our knowledge, few studies have assessed and characterized dietary patterns, broader lifestyle behaviors, and BMI/overweight-obesity specifically in young Romanians aged 18–30 years. To address this gap, the present cross-sectional, questionnaire-based study aimed to describe lifestyle habits, dietary behaviors, and BMI in Romanian young adults aged 18–30 years living in Romania or the Romanian diaspora, and to examine how these lifestyle and dietary factors relate to BMI and to overweight/obesity.

## 2. Materials and Methods

### 2.1. Study Design and Participants

This was an observational, cross-sectional study using a self-administered structured questionnaire to assess lifestyle habits and dietary behaviors among young adults in Romania. The study was approved by the Ethics Committee of UMF Iuliu Hațieganu Cluj-Napoca (approval no. AVZ111, date 29 April 2025). The study adhered to the principles of the Declaration of Helsinki and complied with the General Data Protection Regulation (GDPR).

Participants were eligible if they were aged 18–30 years, were Romanian citizens residing in Romania or the Romanian diaspora, and were capable of providing informed consent. Based on Romania’s total male (*n* = 10,608,827) and female (*n* = 11,130,573) populations [27], a minimum sample of 385 participants for each sex was estimated to provide a 95% confidence level with a 5% margin of error. Participants were recruited through social-media platforms (Facebook, Instagram [Meta Platforms, Inc., Menlo Park, CA, USA]), university student groups, and public announcements. Informed consent was obtained electronically before participants began the questionnaire. Data were collected online via Google Forms between 1 May and 31 July 2025. At the end of the data-collection period, five participants were randomly selected to receive a voucher worth 400 lei.

### 2.2. Questionnaire and Measures

The developed structured questionnaire was divided into three parts (Supplementary data-collection questionnaire). Part 1 (General Information) collected sociodemographic data (sex, age, marital status, region of residence, urban or rural setting, education, household income, occupational activity), self-reported anthropometric data (height, weight), and the presence of self-reported cardiometabolic diagnoses. Part 2 (Lifestyle Assessment) assessed physical-activity level, smoking status, sleep patterns and duration, alcohol-consumption habits, and psychological well-being (work stress, home stress). Part 3 (Nutritional Assessment) covered dietary pattern (e.g., omnivore, vegetarian), consumption frequency of 23 specific food and beverage groups, water intake, meal and snack frequency, food-label reading habits, frequency of cooking at home versus ordering out, eating-alone frequency, and compulsive-eating frequency.

#### 2.2.1. Outcome Variables

The primary outcome was body mass index (BMI), calculated as self-reported weight in kilograms divided by the square of self-reported height in meters (kg/m^2^) and analyzed as a continuous variable. The secondary outcome was overweight/obesity, defined as BMI ≥ 25 kg/m^2^ and analyzed as a binary variable. BMI categories were defined according to the WHO classification: underweight (<18.5 kg/m^2^), normal weight (18.5–24.9 kg/m^2^), overweight (25.0–29.9 kg/m^2^), and obesity (≥30.0 kg/m^2^).

#### 2.2.2. Exposure Variables

Five lifestyle and dietary factors were used as main exposures, based on their level of evidence and biological plausibility linking them with BMI or overweight/obesity, each being derived from a specific questionnaire item. Physical activity was assessed in Part 2, Q1, and categorized as inactive, insufficiently active, or meeting guidelines [28]. Sleep duration was assessed in Part 2, Q5; the original categories <3 h and 3–5 h were collapsed into ≤5 h/night, with 6–8 h/night used as the reference category [29]. Breakfast frequency was assessed in Part 3, Q5 as the weekly frequency of breakfast consumption and categorized as ≤2, 3–4, 5–6, or 7 days/week [30]. Compulsive eating was assessed in Part 3, Q7, using the item ‘Eat compulsively (repetitive, uncontrollable behavior)’ and categorized as <1 time/week, 1–3 times/week, 4–6 times/week, or once/day or more [31]. Soft-drink intake was assessed in Part 3, Q8, using the item ‘Carbonated beverages (Coca-Cola, Pepsi, Sprite, etc.)’ and categorized using the same four frequency levels [13].

### 2.3. Composite Dietary Scores

Three composite dietary indices were computed to serve as a robustness check of the individual-exposure results. Item selection was guided by concordance across established dietary quality frameworks, including the DASH diet score [32], the Healthy Eating Index-2020 [33], the Mediterranean Diet Score [34], and the EAT-Lancet Planetary Health Diet [35]. A healthy-food score was calculated as the unweighted mean of eight food-frequency items: vegetables, fruit, leafy greens, whole grains, legumes, nuts/seeds, fish, and dairy products (each scored 1–4). An unhealthy-food score was calculated as the unweighted mean of seven items: red meat, processed meat, fried foods, sweets, pastry, soft drinks, and refined grains (bread/pasta/cereals; each scored 1–4) [36]. Eggs and white meat were excluded from both indices because current evidence regarding their cardiometabolic effects is mixed or largely neutral [37,38], while red/orange fruit and vegetables were not included in the healthy-food score because they conceptually overlap with the vegetables and fruit items.

A meal-regularity score was defined as the sum of the weekly frequencies of breakfast, lunch, and dinner (range 3–21).

These indices have not been externally validated in our study; their use here is limited to a robustness check of the individual-exposure findings.

### 2.4. Geographic and Additional Variables

The geographic variable ‘region of residence’ was handled as a nominal factor with nine categories corresponding to the eight Romanian NUTS-2 development regions (North-West, Center, North-East, South-East, South-Muntenia, Bucharest-Ilfov, South-West Oltenia, and West) and a Diaspora group. For regression, North-West served as the reference category. Several additional lifestyle and dietary variables were collected for descriptive purposes. Food-consumption frequency was assessed for 23 food and beverage categories, each on a four-level ordinal scale (<1/week, 1–3/week, 4–6/week, ≥1/day). Other variables included dietary pattern (omnivore, vegetarian, vegan, pescetarian, raw vegan), water intake (<1 L/day, 1–2 L/day, >2 L/day), self-reported sleep problems (yes/no), home stress (four categories), food-label reading habits (yes/sometimes/no), eating-alone frequency, and marital status.

### 2.5. Statistical Analysis

All analyses were performed using RStudio 2026.01.2+418 (Posit Software, PBC, Boston, MA, USA). Continuous variables were summarized as mean ± standard deviation (SD) for normally distributed data and median [interquartile range] for count-type variables. Categorical variables were summarized as *n* (%). Sex differences were tested using Welch’s *t*-test or the Wilcoxon rank-sum test for continuous variables and the chi-squared test or Fisher’s exact test for categorical variables.

For the primary outcome (BMI, continuous), multivariable linear regression with heteroscedasticity-consistent (HC3) sandwich standard errors was used [39]. For the secondary outcome (overweight/obesity, binary), modified Poisson regression with robust (HC0) variance estimation was used to estimate prevalence ratios (PRs). Two nested models were fit for each outcome: Model 1 adjusted for age (continuous, per year) and sex (male vs. female) only, and Model 2 (the main inference model) additionally adjusted for smoking (current smoker vs. not), alcohol (ordinal: none, <7 drinks/week, 7–14/week, >14/week), urban/rural residence, education (ordinal: middle school, secondary, university), income (ordinal, six categories from <2000 lei to >10,000 lei), work stress (ordinal: never, sometimes, often, always), and region of residence (nominal factor with nine categories corresponding to the eight Romanian NUTS-2 development regions plus a Diaspora group; North-West serving as the reference category) [40,41,42]. A *P*-for-trend was computed for each exposure by re-fitting it as a numeric ordinal predictor (scored 1, 2, 3, 4). Ordinal food-frequency and lifestyle scales were retained both as labeled factors (for tables) and as 1–4 numeric ordinal scores (for trend tests).

A composite-score model (Model 3) replaced the individual food and lifestyle exposures with the healthy-food score, unhealthy-food score, and meal-regularity score, retaining the same covariates as Model 2. This model served as a robustness check to assess whether summary dietary patterns corroborated the individual-exposure findings. Collinearity was assessed using variance inflation factors (VIFs); the maximum VIF across all terms in Model 2 was 1.17, indicating no collinearity concern. Formal sex × exposure interaction terms were tested for each of the five main exposures using nested-model *F*-tests.

Four sensitivity analyses were conducted: (a) comparison of effect sizes between Model 1 (minimally adjusted) and Model 2 (fully adjusted) to evaluate confounding stability; (b) exclusion of the 73 participants reporting a cardiometabolic diagnosis to assess the potential influence of reverse causation or disease-related dietary changes; (c) re-fitting the main exposures as numeric ordinal scores to assess per-step linearity; and (d) formal education × exposure interaction tests, conducted by dichotomizing education as non-university versus university and testing interaction terms for all five main exposures, to evaluate whether associations generalize across educational strata. In an exploratory analysis, snacks per day were added to the main model covariates.

## 3. Results

### 3.1. Sample Characteristics

After verifying the self-reported information, three observations with implausible anthropometric or dietary values (two heights < 1.40 m [1.15 m and 1.35 m] and one meals/day value = 23) were excluded, leaving an analytic sample of *n* = 1202 participants (mean age 23.2 ± 3.3 years; 46.8% women; 81.4% urban residents) for all analyses (Table 1). Mean BMI was 23.8 ± 4.2 kg/m^2^; 6.7% were classified as underweight, 25.0% as overweight, and 7.4% as obese, yielding an overall overweight/obesity prevalence of 32.4%. University-level education was reported by 77.9% of participants. A cardiometabolic diagnosis was reported by 6.1% of participants. Regarding sleep, most participants reported sleeping 6–8 h/night (78.2%).

Men had significantly higher mean BMI (25.0 vs. 22.5 kg/m^2^, *p* < 0.001) and a markedly higher prevalence of overweight (33.8% vs. 15.1%, *p* < 0.001) and obesity (9.2% vs. 5.3%, *p* = 0.014) than women. Men were more likely to be current smokers (34.6% vs. 22.7%, *p* < 0.001), to meet physical-activity guidelines (56.8% vs. 34.1%, *p* < 0.001), and to consume soft drinks at least four times per week (18.6% vs. 8.9%, *p* < 0.001) (Supplementary Table S1), whereas women reported higher rates of compulsive eating at least once daily (5.7% vs. 3.0%, *p* = 0.029). Regarding the food-consumption profile (Supplementary Table S1), women reported more frequent consumption of vegetables, fruit, dairy, and tea, while men consumed red meat, processed meat, fried foods, and soft drinks more frequently.

### 3.2. Compulsive Eating and Soft-Drink Intake Were Dose-Dependently Associated with Higher BMI

In the fully adjusted linear regression model (Table 2), compulsive eating showed a clear, graded association with BMI. Relative to the reference category (<1/week), participants reporting compulsive eating 1–3 times per week had a BMI that was 1.15 kg/m^2^ higher (β = 1.15, 95% CI 0.60 to 1.69, *p* < 0.001), those reporting 4–6 times per week had a BMI 2.60 kg/m^2^ higher (β = 2.60, 95% CI 1.59 to 3.61, *p* < 0.001), and those reporting daily or more frequent compulsive eating had a BMI 3.00 kg/m^2^ higher (β = 3.00, 95% CI 1.38 to 4.62, *p* < 0.001). The test for linear trend was highly significant (*P*-trend < 0.001), indicating that each step up in compulsive-eating frequency was associated with an approximately 1.15 kg/m^2^ increment in BMI.

Soft-drink intake showed a similar dose–response pattern. Compared with infrequent consumption (<1/week), intake of 1–3 times per week was associated with a 0.55 kg/m^2^ higher BMI (β = 0.55, 95% CI 0.01 to 1.09, *p* = 0.045), intake 4–6 times per week with a 1.05 kg/m^2^ higher BMI (β = 1.05, 95% CI 0.25 to 1.84, *p* = 0.010), and daily or more frequent intake with a 1.29 kg/m^2^ higher BMI (β = 1.29, 95% CI 0.39 to 2.19, *p* = 0.005). The *P*-trend was <0.001.

**Figure 1:**
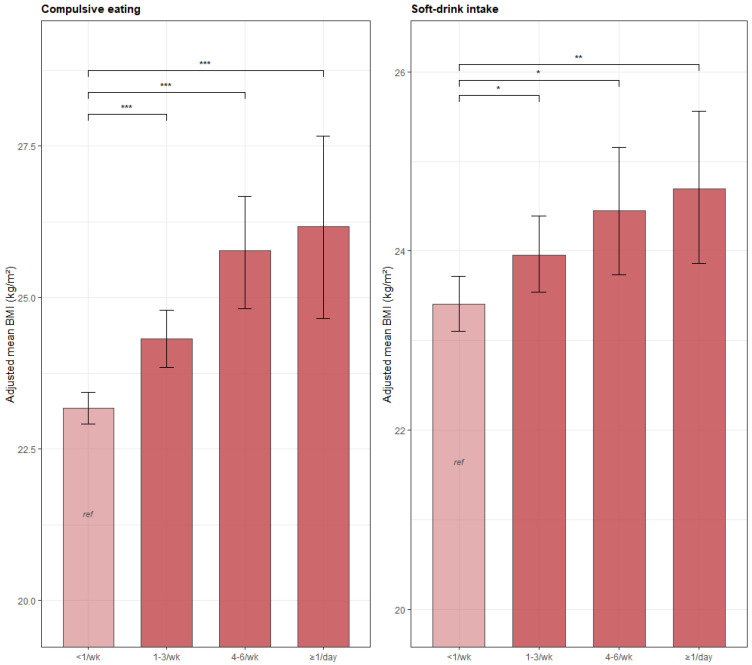
Adjusted mean BMI (95% CI) by frequency of compulsive eating and soft-drink intake. Bars represent marginally standardized adjusted means; error bars are 95% CIs (1000 bootstrap replicates). Adjusted for age, sex, smoking, alcohol, residence, education, income, work stress, and region. * *p* < 0.05, ** *p* < 0.01, *** *p* < 0.001 vs. reference category.

Physical activity, breakfast frequency, and sleep duration showed no statistically significant associations with continuous BMI after full covariate adjustment (all *p* > 0.12). Current smoking was associated with a modestly higher BMI (β = 0.73, 95% CI 0.14 to 1.33, *p* = 0.016) (Supplementary Table S2). The dose–response patterns for compulsive eating and soft-drink intake are illustrated visually in Figure 1.

### 3.3. Prevalence Ratios for Overweight/Obesity Confirmed the Main Associations and Revealed Additional Threshold Effects

The modified Poisson regression for overweight/obesity (BMI ≥ 25; Table 3) confirmed the dose–response associations observed in the continuous model. For compulsive eating, the prevalence ratio increased from 1.24 (95% CI 1.03 to 1.48, *p* = 0.020) at 1–3/week to 1.73 (95% CI 1.36 to 2.19, *p* < 0.001) at 4–6/week and 1.86 (95% CI 1.35 to 2.57, *p* < 0.001) at ≥1/day, relative to <1/week. For soft-drink intake, prevalence ratios increased from 1.27 (95% CI 1.04 to 1.55) at 1–3/week to 1.42 (95% CI 1.11 to 1.82) at ≥1/day. The consistency between the continuous and binary models supports the robustness of these two associations.

**Figure 2:**
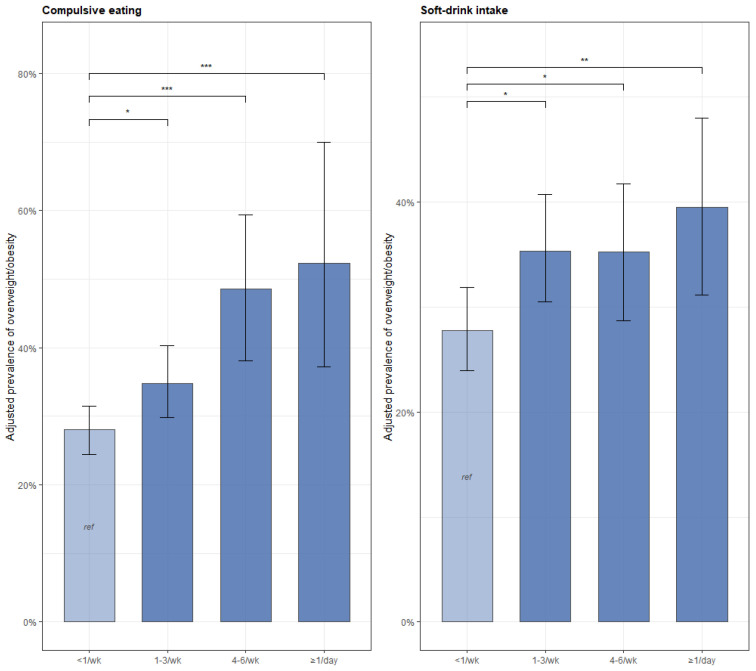
Adjusted prevalence of overweight/obesity (95% CI) by frequency of compulsive eating and soft-drink intake. Bars represent marginally standardized adjusted prevalences from modified Poisson regression; error bars are 95% CIs (1000 bootstrap replicates). Same covariate adjustment as Figure 1. * *p* < 0.05, ** *p* < 0.01, *** *p* < 0.001 vs. reference category.

The binary outcome model also revealed two threshold effects that were not apparent in the continuous-BMI analysis. Daily breakfast consumption was associated with a 25% lower prevalence of overweight/obesity compared with rare breakfast (≤2 days/week; PR = 0.75, 95% CI 0.61 to 0.92, *p* = 0.007), whereas intermediate breakfast frequencies (3–4/week, 5–6/week) were not significantly different from the reference. Short sleep (≤5 h vs. 6–8 h) was associated with a 34% higher prevalence of overweight/obesity (PR = 1.34, 95% CI 1.05 to 1.69, *p* = 0.016). One plausible explanation for these threshold effects is that these behaviors shift individuals across a clinical cut-point rather than shifting the population mean. The adjusted prevalence patterns are illustrated in Figure 2.

### 3.4. Composite Dietary Scores Support the Main Findings

In the composite-score model (Table 4), the unhealthy-food score showed a positive association with continuous BMI (β = 0.42, 95% CI −0.03 to 0.87, *p* = 0.065) and overweight/obesity prevalence (PR = 1.13, 95% CI 0.99 to 1.29, *p* = 0.062), with confidence intervals narrowly including the null. The meal-regularity score was inversely associated with both BMI (β = −0.08, 95% CI −0.14 to −0.02, *p* = 0.008) and overweight/obesity (PR = 0.97, 95% CI 0.95 to 0.98, *p* < 0.001). The healthy-food score was not significantly associated with either outcome. The direction of these associations is consistent with the individual-exposure findings. The full model is presented in Supplementary Table S3.

### 3.5. Robustness and Sensitivity

Effect sizes were stable between the minimally adjusted model (age + sex only) and the fully adjusted model (Supplementary Table S2), indicating that confounding by sociodemographic and lifestyle covariates was modest. None of the five sex × exposure interactions reached statistical significance (all *p* > 0.13; Supplementary Table S4), justifying the use of pooled estimates. Excluding the 73 participants with a cardiometabolic diagnosis did not change the direction or significance of any main association (Supplementary Table S5). Re-fitting the main exposures as numeric ordinal scores yielded consistent per-step effect sizes (e.g., compulsive eating β = 1.15 per ordinal step, soft drinks β = 0.46 per step; *p* < 0.001).

Similarly, none of the five education × exposure interactions reached significance for soft-drink intake (*p* = 0.406), compulsive eating (*p* = 0.212), breakfast frequency (*p* = 0.232), or physical activity (*p* = 0.165), indicating that the main associations did not differ between non-university and university-educated participants. Only the sleep duration × education interaction was nominally significant (*p* = 0.029) (Supplementary Tables S6). In education-stratified models, the direction and magnitude of the soft-drink and compulsive-eating associations were consistent across both strata, although confidence intervals were wider in the non-university group (*n* = 266) as expected (Supplementary Tables S7).

In the exploratory model that added snacks per day to the main covariates, each additional daily snack was inversely associated with BMI (β = −0.60, 95% CI −0.85 to −0.35, *p* < 0.001) and overweight/obesity (PR = 0.82, 95% CI 0.75 to 0.89, *p* < 0.001). However, this finding should be interpreted cautiously because self-reported snack frequency is susceptible to measurement artifact and may not represent a causal pathway.

## 4. Discussion

In this cross-sectional study of 1202 Romanian young adults aged 18–30 years, overweight or obesity was present in roughly one-third of the sample and was more common in men than in women, consistent with a broader pattern of sex-stratified adiposity in Romania and in the wider WHO European Region [1,2,3]. Two exposures emerged as robust, dose-dependent correlates of adiposity. Self-reported compulsive eating was associated with higher mean BMI and with a graded increase in the prevalence of overweight/obesity across frequency categories. Soft-drink intake also showed a dose–response pattern for both outcomes. Daily breakfast consumption was associated with a lower prevalence of overweight/obesity, but not with continuous BMI, and short sleep duration (≤5 h/night) showed the same threshold-type association only with the binary outcome. Composite-score analyses were directionally consistent with the main findings: the unhealthy-food score showed a positive trend with BMI and overweight/obesity, and meal regularity was inversely associated with both outcomes. Importantly, several associations were not only statistically significant but also potentially meaningful in magnitude: daily compulsive eating was associated with an approximately 3.0 kg/m^2^ higher BMI, daily soft-drink intake with an approximately 1.3 kg/m^2^ higher BMI, daily breakfast consumption with a 25% lower prevalence of overweight/obesity, and short sleep duration with a 34% higher prevalence. These estimates should be interpreted cautiously but may represent relevant behavioral patterns for public-health prevention.

Sensitivity analyses, sex-by-exposure interactions, and education-by-exposure interactions were consistent and non-significant for the two primary exposures, supporting the stability and generalizability of the pooled estimates across demographic subgroups.

### 4.1. Comparison with Romanian Literature

The BMI profile observed here aligns with the available Romanian evidence. In the nationally representative PREDATORR study, the age- and sex-adjusted prevalence of obesity in adults aged 20–79 years was 31.9% and that of overweight 34.7% [23]; in the general-population ORO study, the prevalence of overweight and obesity in the 18–39-year age group was 31.1% and 9.9%, respectively [24]. Our overweight/obesity prevalence of 32.4% in 18–30-year-olds is broadly consistent with these earlier estimates for younger Romanian adults, while the higher male burden mirrors data from PREDATORR [23] and from post-pandemic Romanian surveys of dietary and lifestyle quality [43]. However, an important caveat is that BMI, while practical, does not distinguish between fat mass and lean mass and cannot identify individuals with normal weight obesity (NWO), a condition defined by normal BMI but elevated body fat percentage, which is associated with increased cardiometabolic risk, systemic inflammation, and cardiovascular mortality [44]. NWO may be particularly relevant in young adults, in whom adverse body composition can develop before BMI reaches overweight thresholds [45]. Because our study relied on self-reported height and weight without body composition assessment, an unknown proportion of participants classified as normal-weight may in fact have had excess adiposity. This means that the true burden of adiposity-related risk in this sample is likely underestimated, and that associations between lifestyle exposures and body fat may be stronger than those captured by BMI alone.

Sex differences in dietary patterns in our data, i.e., men reporting more frequent red and processed meat, fried foods, and soft drinks, and women reporting more frequent fruit, vegetable, and dairy consumption, also replicate the sex-stratified patterns described in the ORO cohort, in which a ‘High meat/High fat’ pattern was associated with male sex and higher odds of obesity, while a ‘Prudent’ pattern was associated with female sex and lower odds of obesity [46].

Romanian evidence on dietary behaviors specifically in young adults remains limited but is broadly consistent with the direction of our findings. In a repeated cross-sectional survey of university students in Cluj-Napoca conducted in 2003 and 2016, Lotrean et al. identified recurring dietary patterns, with a healthier pattern associated with lower overweight and with higher physical activity, and noted persistent sex differences in weight-related behaviors [47]. In a large survey of Romanian young adults aged 18–30 years, Rada C. found that eating habits clustered meaningfully with BMI categories, even though dietary knowledge did not consistently translate into healthier weight status [48]. Among Romanian medical students, mindful eating was independently associated with a lower likelihood of excess weight, whereas nutrition knowledge was not [49], suggesting that eating behaviors, including disinhibited or compulsive patterns, may be at least as relevant as nutrition knowledge in relation to weight status.

Earlier work by Mocanu V. described disturbed eating attitudes in Romanian college students, particularly in women, showing that there is a high prevalence of disturbed eating attitudes and behaviors [50]. Compared with students who did not report dieting behavior, those who did were more likely to engage in excessive exercise and to consume less cereals and meat, while eating more legumes. More recent adult studies from Western Romania showed that dietary habits and behavioral factors differ systematically between normal-weight adults and those with overweight or obesity [51,52]. Romanian pediatric and adolescent data, including studies from Bucharest [53], North-Western Romania [54], and Transylvania [26,55], also identified high intake of soft drinks, sweets, and fast food, along with low fruit and vegetable consumption, as key correlates of unfavorable nutritional status;

### 4.2. Comparison with the Wider Literature

Our soft-drink findings are consistent with a well-established wider literature. The systematic review and meta-analysis by Malik et al. demonstrated a positive association between sugar-sweetened beverage intake and weight gain in both children and adults [14], and an updated synthesis reached similar conclusions [13]. The dose–response gradient in BMI and overweight/obesity observed here in Romanian young adults is similar to results reported in adult cohorts in Western Europe and North America [13,14].

Also, our compulsive-eating findings extend a growing literature linking disinhibited, emotional, and binge-type eating to higher BMI. Dakanalis et al. reported consistent associations between emotional eating and overweight/obesity [15], and a meta-analysis by Vasileiou and Abbott found significantly higher levels of emotional eating in adults with obesity than in those with a healthy weight [16]. Breakfast skipping has likewise been associated with an increased risk of overweight and obesity in observational meta-analyses [17,30], and short sleep duration, especially ≤5 h, has been linked to higher obesity risk in meta-analytic estimates across adult populations [56,57]. Findings from Nelson et al. showed that emerging adulthood is a critical period in which these behavioral risk factors cluster and shape later adulthood [9].

The exploratory inverse association between snacks/day and adiposity should be interpreted cautiously, because the wider literature on snacking frequency and weight status in adults is inconsistent. For instance, the 2025 Dietary Guidelines Advisory Committee systematic review concluded that overall snacking in adults may not be associated with body composition or obesity risk, whereas evening snacking may be less favorable, suggesting that snack timing, quality, and contextual eating patterns may matter more than snack count alone [58]. The results are also highly sensitive to how a ‘snack’ is defined. In a large NHANES analysis, most associations between snack frequency and adiposity were null once alternative snack definitions were applied [59].

Within the Central and Eastern Europe (CEE) context, the behavioral patterns observed in our sample are not unique to Romania. Obesity prevalence rates in CEE countries are generally higher than in Western and Northern Europe, a pattern attributed in part to post-socialist dietary transitions, increased availability of processed foods, and more sedentary lifestyles [60,61]. In Poland, emotional eating was reported by 37.9% of students and was associated with problems in estimating portion size and caloricity of meals [62], while a representative Polish adult survey identified sex-stratified differences in weight-related behaviors that parallel the male-predominant obesogenic pattern in our data [63]. In Czechia, with one of the highest overweight prevalences in Europe (over 60% of adults), soft-drink consumption and sedentary behavior have been identified as key modifiable risk factors [64]. These cross-national parallels suggest that the associations reported here may reflect shared environmental and transitional influences across CEE populations, although direct cross-country comparisons using harmonized methods are needed.

### 4.3. Interpretation

Several mechanisms might potentially explain the observed associations. Soft-drink intake provides substantial energy with low satiety value and may promote positive energy balance, and tends to cluster with other energy-dense food choices, so that frequent soft-drink intake likely behaves both as a direct caloric exposure and as a marker of a broader obesogenic dietary pattern [13,14].

Compulsive eating may reflect a loss-of-control eating phenotype, in which eating is driven less by physiological hunger and more by impaired inhibitory control, emotional distress, stress reactivity, reward sensitivity, and exposure to highly palatable, energy-dense foods. These mechanisms may promote higher total energy intake and difficulty stopping eating once initiated, thereby helping to explain the observed association with higher BMI and overweight/obesity [15,16,65,66]. However, because compulsive eating was assessed only by self-reported frequency, our study could not distinguish between episode frequency, episode size, emotional triggers, or subjective distress.

The observation that unhealthy-food and meal-regularity composite scores showed associations in the expected directions is consistent with the hypothesis that overall dietary structure, rather than any single food, contributes to the observed associations, a pattern already described in Romanian adults in the ORO study [46].

Several findings deserve careful interpretation. In our analysis, physical activity was not independently associated with continuous BMI after full adjustment; however, this finding should not be interpreted as evidence that physical activity is unrelated to adiposity. It is well-documented that aerobic exercise reduces body weight, waist circumference, and body fat in adults with overweight or obesity. Particularly, ≥150 min/week of moderate-to-vigorous activity was associated with clinically important reductions in waist circumference and body fat [67], changes that are themselves associated with improved cardiometabolic risk profiles [68]. The null association observed in our study may be explained by measurement sensitivity, as physical activity was assessed using a single self-reported item rather than a validated multi-item instrument or objective activity measure. As shown by Lim et al., self-reported physical activity differed from accelerometer-derived activity, and correction for measurement error substantially deattenuated associations with obesity and diabetes, suggesting that imprecise self-report may bias associations toward the null [69].

Daily breakfast consumption and short sleep were associated with overweight/obesity but not with continuous BMI: a pattern consistent with threshold effects, in which these behaviors shift individuals across the 25 kg/m^2^ cut-point rather than the population mean. Smoking, which was associated with modestly higher BMI in our fully adjusted model, also warrants consideration as a contributor to adverse body composition beyond what BMI captures. In a cross-sectional study of Slovak young adults aged 19–30 years, a population broadly comparable to ours, Falbová et al. found that regular smokers had significantly higher waist circumference, body fat percentage, trunk fat mass, and visceral fat area than non-smokers, and that fat mass increased with smoking frequency [70]. These findings suggested that the positive association between current smoking and BMI observed in our data may partly reflect smoking-related central adiposity.

Finally, the absence of significant sex-by-exposure interactions, despite pronounced descriptive sex differences in behaviors and BMI, suggests that the magnitude of the associations between lifestyle exposures and adiposity is broadly similar in men and women, while the prevalence of the exposures themselves differs, a pattern that justifies pooled estimation without stratified presentation.

### 4.4. Future Directions

Longitudinal studies are needed to clarify the temporal relationship between soft-drink intake, self-reported compulsive eating, physical activity, and weight trajectories in Romanian young adults. Future research should use measured anthropometry and validated instruments for dietary intake, eating behavior, and physical activity, such as validated food-frequency questionnaires, the Three-Factor Eating Questionnaire, the Binge Eating Scale, or validated physical-activity questionnaires/objective activity measures. Such assessments would help determine whether the behavioral patterns observed here reflect robust risk factors or are partly explained by measurement error from single-item self-report measures.

### 4.5. Strengths and Limitations

This study has several important strengths, alongside limitations that should be considered when interpreting the findings. To our knowledge, it is among the largest recent cross-sectional surveys focused specifically on Romanian young adults aged 18–30 years, a group under-represented in prior Romanian research [23,24,26,47,50,53,54,71,72]. Recruitment from all eight NUTS-2 regions and the diaspora provided broad geographic coverage, while multiple lifestyle domains were assessed simultaneously, and the analytical approach captured both continuous BMI differences and overweight/obesity prevalence, with additional composite-score, sex-interaction, and sensitivity analyses supporting robustness.

Nonetheless, several limitations should be acknowledged. First, the cross-sectional design prevents causal inference and allows the possibility of reverse causation. Second, the questionnaire was not formally validated, and several key exposures, including physical activity, sleep duration, compulsive eating, and soft-drink intake, were assessed using single self-reported items rather than validated multi-item instruments. This may have introduced measurement error and exposure misclassification, potentially affecting the magnitude of the observed associations. Third, all data were self-reported, which may have led to social-desirability bias and underestimation of BMI. Critically, BMI does not distinguish between fat mass and lean mass, and our study could not identify individuals with normal weight obesity, those with normal BMI but elevated body fat, who may carry unrecognized cardiometabolic risk. The absence of body composition measures (e.g., bioelectrical impedance, waist circumference) is a meaningful limitation that may have led to misclassification of adiposity status and attenuation of the observed associations. Finally, convenience sampling through social media and university networks over-represented urban, university-educated, and digitally connected participants. Although formal education × exposure interaction tests showed that the associations of compulsive eating and soft-drink intake with BMI did not differ significantly between non-university and university-educated participants, the small size of the non-university subgroup (*n* = 266) limits the power of this comparison, and the findings may not generalize to populations with lower educational attainment or different socioeconomic profiles. The composite dietary indices used in this study were constructed for robustness-checking purposes, informed by but not identical to validated indices such as the DASH diet score or HEI-2020; they have not been externally validated and should be interpreted accordingly.

## 5. Conclusions

In this cross-sectional study of Romanian young adults aged 18–30 years, self-reported compulsive eating and soft-drink intake were the behavioral exposures most consistently associated with higher BMI and a higher prevalence of overweight/obesity in fully adjusted models. Daily breakfast consumption and adequate sleep duration (≥6 h) were associated with lower overweight/obesity prevalence, while physical activity was not independently associated after adjustment. These findings should be interpreted as hypothesis-generating rather than causal, given the cross-sectional design, reliance on self-reported data from a non-validated questionnaire, and the use of BMI without direct body composition assessment. Men showed a higher prevalence of overweight/obesity and a more unfavorable dietary profile than women. The results help address a gap in contemporary Romanian data on diet, lifestyle, and adiposity in young adults and suggest that compulsive eating and soft-drink intake merit attention as potential intervention targets. Longitudinal studies with validated instruments and measured anthropometry, including body composition, are needed to confirm these associations and to assess whether targeted behavioral interventions can reduce adiposity in this population.

## Figures and Tables

**Table 1:** Baseline characteristics of the analytic sample (*n* = 1202).

Variable	Level	Overall (*n* = 1202)	Female (*n* = 563)	Male (*n* = 639)	P (Overall)	P (Level)
**A. Sociodemographic & socioeconomic**						
Age (years)		23.2 ± 3.3	23.3 ± 3.2	23.1 ± 3.3	0.266	
Sex		*n* = 1202	*n* = 563 (46.8%)	*n* = 639 (53.2%)		
Region of residence					<0.001	
	North-West	586 (48.8%)	218 (38.7%)	368 (57.6%)		<0.001
	Center	229 (19.1%)	98 (17.4%)	131 (20.5%)		0.197
	Bucharest-Ilfov	102 (8.5%)	82 (14.6%)	20 (3.1%)		<0.001
	North-East	102 (8.5%)	67 (11.9%)	35 (5.5%)		<0.001
	West	60 (5.0%)	36 (6.4%)	24 (3.8%)		0.050
	South-East	40 (3.3%)	26 (4.6%)	14 (2.2%)		0.029
	Diaspora	30 (2.5%)	10 (1.8%)	20 (3.1%)		0.188
	South-Muntenia	29 (2.4%)	16 (2.8%)	13 (2.0%)		0.470
	South-West Oltenia	24 (2.0%)	10 (1.8%)	14 (2.2%)		0.759
Urban/rural					0.421	
	Urban	978 (81.4%)	464 (82.4%)	514 (80.4%)		0.421
	Rural	224 (18.6%)	99 (17.6%)	125 (19.6%)		0.421
Marital status					<0.001	
	Married or in a stable relationship	324 (27.0%)	210 (37.3%)	114 (17.8%)		<0.001
	Single	878 (73.0%)	353 (62.7%)	525 (82.2%)		<0.001
Education					<0.001	
	Middle school	4 (0.3%)	1 (0.2%)	3 (0.5%)		0.627
	Secondary	262 (21.8%)	83 (14.7%)	179 (28.0%)		<0.001
	University	936 (77.9%)	479 (85.1%)	457 (71.5%)		<0.001
Occupational activity					<0.001	
	Sedentary (desk)	900 (74.9%)	455 (80.8%)	445 (69.6%)		<0.001
	Standing/walking	208 (17.3%)	101 (17.9%)	107 (16.7%)		0.638
	Physically demanding	94 (7.8%)	7 (1.2%)	87 (13.6%)		<0.001
Household income					<0.001	
	<2000 lei	234 (19.5%)	124 (22.0%)	110 (17.2%)		0.042
	2000–4000	267 (22.2%)	150 (26.6%)	117 (18.3%)		<0.001
	4000–6000	233 (19.4%)	104 (18.5%)	129 (20.2%)		0.498
	6000–8000	168 (14.0%)	68 (12.1%)	100 (15.6%)		0.089
	8000–10,000	121 (10.1%)	54 (9.6%)	67 (10.5%)		0.676
	>10,000	179 (14.9%)	63 (11.2%)	116 (18.2%)		<0.001
**B. Anthropometric & clinical**						
Height (cm)		173.0 ± 9.8	165.3 ± 6.2	179.8 ± 7.0	<0.001	
Weight (kg)		71.8 ± 16.2	61.5 ± 11.8	80.8 ± 14.0	<0.001	
BMI (kg/m^2^)		23.8 ± 4.2	22.5 ± 4.0	25.0 ± 4.1	<0.001	
BMI category					<0.001	
	Underweight (<18.5)	80 (6.7%)	64 (11.4%)	16 (2.5%)		<0.001
	Normal weight (18.5–24.9)	732 (60.9%)	384 (68.2%)	348 (54.5%)		<0.001
	Overweight (25.0–29.9)	301 (25.0%)	85 (15.1%)	216 (33.8%)		<0.001
	Obesity (≥30.0)	89 (7.4%)	30 (5.3%)	59 (9.2%)		0.014
Cardiometabolic disease					0.199	
	No	1129 (93.9%)	523 (92.9%)	606 (94.8%)		0.199
	Yes	73 (6.1%)	40 (7.1%)	33 (5.2%)		0.199
**C. Lifestyle**						
Physical activity					<0.001	
	Inactive	112 (9.3%)	68 (12.1%)	44 (6.9%)		0.003
	Insufficient	535 (44.5%)	303 (53.8%)	232 (36.3%)		<0.001
	Meeting guidelines	555 (46.2%)	192 (34.1%)	363 (56.8%)		<0.001
Smoking status					<0.001	
	Never	740 (61.6%)	378 (67.1%)	362 (56.7%)		<0.001
	Ex (>6 mo)	71 (5.9%)	38 (6.7%)	33 (5.2%)		0.298
	Ex (<6 mo)	42 (3.5%)	19 (3.4%)	23 (3.6%)		0.957
	Current	349 (29.0%)	128 (22.7%)	221 (34.6%)		<0.001
Sleep problems					<0.001	
	No	785 (65.3%)	340 (60.4%)	445 (69.6%)		<0.001
	Yes	417 (34.7%)	223 (39.6%)	194 (30.4%)		<0.001
Sleep duration					0.397	
	≤5 h	110 (9.2%)	57 (10.1%)	53 (8.3%)		0.318
	6–8 h	940 (78.2%)	431 (76.6%)	509 (79.7%)		0.219
	>8 h	152 (12.6%)	75 (13.3%)	77 (12.1%)		0.565
Alcohol intake					<0.001	
	None	652 (54.2%)	374 (66.4%)	278 (43.5%)		<0.001
	<7/week	468 (38.9%)	180 (32.0%)	288 (45.1%)		<0.001
	7–14/week	70 (5.8%)	8 (1.4%)	62 (9.7%)		<0.001
	>14/week	12 (1.0%)	1 (0.2%)	11 (1.7%)		0.017
Work stress					<0.001	
	Never	196 (16.3%)	73 (13.0%)	123 (19.2%)		0.004
	Sometimes	493 (41.0%)	191 (33.9%)	302 (47.3%)		<0.001
	Often	418 (34.8%)	236 (41.9%)	182 (28.5%)		<0.001
	Always	95 (7.9%)	63 (11.2%)	32 (5.0%)		<0.001
Home stress					<0.001	
	Never	305 (25.4%)	102 (18.1%)	203 (31.8%)		<0.001
	Sometimes	631 (52.5%)	302 (53.6%)	329 (51.5%)		0.491
	Often	229 (19.1%)	134 (23.8%)	95 (14.9%)		<0.001
	Always	37 (3.1%)	25 (4.4%)	12 (1.9%)		0.016
**D. Eating habits & food-related behaviors**						
Dietary pattern					0.065	
	Omnivore	1143 (95.1%)	526 (93.4%)	617 (96.6%)		0.018
	Pescetarian	22 (1.8%)	14 (2.5%)	8 (1.3%)		0.168
	Raw vegan	1 (0.1%)	0 (0.0%)	1 (0.2%)		1.000
	Vegan	19 (1.6%)	13 (2.3%)	6 (0.9%)		0.095
	Vegetarian	17 (1.4%)	10 (1.8%)	7 (1.1%)		0.452
Water intake					<0.001	
	<1 L/day	164 (13.6%)	118 (21.0%)	46 (7.2%)		<0.001
	1–2 L/day	669 (55.7%)	352 (62.5%)	317 (49.6%)		<0.001
	>2 L/day	369 (30.7%)	93 (16.5%)	276 (43.2%)		<0.001
Meals per day		3.0 [2.0–3.0]	3.0 [2.0–3.0]	3.0 [2.0–3.0]	0.049	
Snacks per day		2.0 [1.0–2.0]	2.0 [1.0–2.0]	2.0 [1.0–2.0]	0.997	
Breakfast (days/week)		5.0 [3.0–7.0]	5.0 [3.0–7.0]	5.0 [2.0–7.0]	<0.001	
Lunch (days/week)		7.0 [5.0–7.0]	7.0 [5.0–7.0]	7.0 [5.0–7.0]	0.792	
Dinner (days/week)		7.0 [5.0–7.0]	7.0 [5.0–7.0]	7.0 [5.0–7.0]	0.335	
Reads food labels					<0.001	
	No	174 (14.5%)	53 (9.4%)	121 (18.9%)		<0.001
	Sometimes	591 (49.2%)	280 (49.7%)	311 (48.7%)		0.756
	Yes	437 (36.4%)	230 (40.9%)	207 (32.4%)		0.003
Cooks at home					<0.001	
	<1/week	138 (11.5%)	34 (6.0%)	104 (16.3%)		<0.001
	1–3/week	378 (31.4%)	159 (28.2%)	219 (34.3%)		0.029
	4–6/week	387 (32.2%)	203 (36.1%)	184 (28.8%)		0.009
	≥1/day	299 (24.9%)	167 (29.7%)	132 (20.7%)		<0.001
Orders out					<0.001	
	<1/week	722 (60.1%)	389 (69.1%)	333 (52.1%)		<0.001
	1–3/week	382 (31.8%)	146 (25.9%)	236 (36.9%)		<0.001
	4–6/week	78 (6.5%)	21 (3.7%)	57 (8.9%)		<0.001
	≥1/day	20 (1.7%)	7 (1.2%)	13 (2.0%)		0.399
Eats alone					0.488	
	<1/week	239 (19.9%)	117 (20.8%)	122 (19.1%)		0.509
	1–3/week	361 (30.0%)	177 (31.4%)	184 (28.8%)		0.350
	4–6/week	303 (25.2%)	138 (24.5%)	165 (25.8%)		0.649
	≥1/day	299 (24.9%)	131 (23.3%)	168 (26.3%)		0.253
Compulsive eating					0.031	
	<1/week	739 (61.5%)	334 (59.3%)	405 (63.4%)		0.167
	1–3/week	314 (26.1%)	143 (25.4%)	171 (26.8%)		0.638
	4–6/week	98 (8.2%)	54 (9.6%)	44 (6.9%)		0.109
	≥1/day	51 (4.2%)	32 (5.7%)	19 (3.0%)		0.029
**E. Derived dietary & lifestyle scores**						
Healthy-food score (1–4)		2.3 ± 0.6	2.4 ± 0.5	2.2 ± 0.6	<0.001	
Unhealthy-food score (1–4)		2.1 ± 0.6	2.0 ± 0.5	2.2 ± 0.6	<0.001	
Meal regularity score (3–21)		16.3 ± 4.1	16.7 ± 3.8	16.0 ± 4.3	0.003	

**Table 2:** Adjusted associations of pre-specified exposures with BMI (linear regression, Model 2). Adjusted for age, sex, smoking, alcohol, urban/rural residence, education, income, work stress, and region. P-trend from numeric ordinal recoding.

Term	β (95% CI)	P	P-Trend
PA: Insufficient (vs. Inactive)	−0.41 (−1.46 to 0.65)	0.448	0.934
PA: Meeting guidelines (vs. Inactive)	−0.20 (−1.27 to 0.87)	0.714	
Sleep: ≤5 h (vs. 6–8 h)	0.79 (−0.22 to 1.80)	0.124	0.197
Sleep: >8 h (vs. 6–8 h)	−0.02 (−0.63 to 0.59)	0.952	
Soft drinks: 1–3/week (vs. <1/week)	0.55 (0.01 to 1.09)	0.045	<0.001
Soft drinks: 4–6/week (vs. <1/week)	1.05 (0.25 to 1.84)	0.010	
Soft drinks: ≥1/day (vs. <1/week)	1.29 (0.39 to 2.19)	0.005	
Compulsive eating: 1–3/week (vs. <1/week)	1.15 (0.60 to 1.69)	<0.001	<0.001
Compulsive eating: 4–6/week (vs. <1/week)	2.60 (1.59 to 3.61)	<0.001	
Compulsive eating: ≥1/day (vs. <1/week)	3.00 (1.38 to 4.62)	<0.001	
Breakfast: 3–4/week (vs. ≤2/week)	−0.02 (−0.76 to 0.72)	0.967	0.316
Breakfast: 5–6/week (vs. ≤2/week)	−0.19 (−0.90 to 0.52)	0.599	
Breakfast: Daily (vs. ≤2/week)	−0.28 (−0.92 to 0.36)	0.389	
Age (per year)	0.26 (0.18 to 0.34)	<0.001	
Male (vs. Female)	2.25 (1.72 to 2.77)	<0.001	

**Table 3:** Adjusted prevalence ratios for overweight/obesity (BMI ≥ 25), modified Poisson regression, Model 2. Same covariate adjustment as Table 2.

Term	PR (95% CI)	P
PA: Insufficient (vs. Inactive)	0.94 (0.73 to 1.20)	0.597
PA: Meeting guidelines (vs. Inactive)	0.94 (0.73 to 1.21)	0.612
Sleep: ≤5 h (vs. 6–8 h)	1.34 (1.05 to 1.69)	0.016
Sleep: >8 h (vs. 6–8 h)	1.03 (0.81 to 1.31)	0.811
Soft drinks: 1–3/week (vs. <1/week)	1.27 (1.04 to 1.55)	0.017
Soft drinks: 4–6/week (vs. <1/week)	1.27 (1.01 to 1.60)	0.044
Soft drinks: ≥1/day (vs. <1/week)	1.42 (1.11 to 1.82)	0.005
Compulsive eating: 1–3/week (vs. <1/week)	1.24 (1.03 to 1.48)	0.020
Compulsive eating: 4–6/week (vs. <1/week)	1.73 (1.36 to 2.19)	<0.001
Compulsive eating: ≥1/day (vs. <1/week)	1.86 (1.35 to 2.57)	<0.001
Breakfast: 3–4/week (vs. ≤2/week)	0.90 (0.73 to 1.11)	0.313
Breakfast: 5–6/week (vs. ≤2/week)	0.89 (0.71 to 1.11)	0.297
Breakfast: Daily (vs. ≤2/week)	0.75 (0.61 to 0.92)	0.007
Age (per year)	1.07 (1.04 to 1.09)	<0.001
Male (vs. Female)	1.91 (1.56 to 2.34)	<0.001

**Table 4:** Composite dietary-score associations with BMI and overweight (OW)/obesity (OB) (Model 3). Adjusted for the same covariates as Model 2. Full model in Supplementary Table S3.

Term	β (95% CI)	P (BMI)	PR (95% CI)	P (OW/OB)
Healthy-food score	−0.11 (−0.54 to 0.32)	0.617	0.92 (0.79 to 1.06)	0.250
Unhealthy-food score	0.42 (−0.03 to 0.87)	0.065	1.13 (0.99 to 1.29)	0.062
Meal regularity score	−0.08 (−0.14 to −0.02)	0.008	0.97 (0.95 to 0.98)	<0.001
Age (per year)	0.24 (0.16 to 0.32)	<0.001	1.06 (1.04 to 1.09)	<0.001
Male (vs. Female)	2.24 (1.74 to 2.75)	<0.001	1.85 (1.52 to 2.25)	<0.001

## Data Availability

The authors confirm that the data supporting the findings of this study are available upon request.

## Supplementary Materials

The following supporting information can be downloaded at: https://www.mdpi.com/article/10.3390/nu18101644/s1, Supplementary data-collection questionnaire: The developed structured questionnaire used to assess variables of interest. Table S1. Food-consumption profile, overall and by sex; Table S2. Full composite-score models (Model 3); Table S3. Full regression results comparing Model 1 and Model 2; Table S4. Sex × exposure interaction tests; Table S5. Sensitivity analyses for BMI and overweight/obesity; Table S6. Education × exposure interaction tests; Table S7. Education-stratified sensitivity analysis for soft-drink intake and compulsive eating.

## References

[1] Phelps N.H., Singleton R.K., Zhou B., Heap R.A., Mishra A., Bennett J.E., Paciorek C.J., Lhoste V.P., Carrillo-Larco R.M., Stevens G.A. (2024). Worldwide Trends in Underweight and Obesity from 1990 to 2022: A Pooled Analysis of 3663 Population-Representative Studies with 222 Million Children, Adolescents, and Adults. Lancet.

[2] Levesque R.J.R. Obesity and Overweight. https://www.who.int/news-room/fact-sheets/detail/obesity-and-overweight.

[3] World Health Organization Regional Office for Europe (2022). WHO European Regional Obesity Report 2022. https://iris.who.int/server/api/core/bitstreams/e65ae612-3723-4df5-a0e3-639dcdaa9ef8/content.

[4] Schnurr T.M., Jakupović H., Carrasquilla G.D., Ängquist L., Grarup N., Sørensen T.I.A., Tjønneland A., Overvad K., Pedersen O., Hansen T. (2020). Obesity, Unfavourable Lifestyle and Genetic Risk of Type 2 Diabetes: A Case-Cohort Study. Diabetologia.

[5] Sun M., da Silva M., Bjørge T., Fritz J., Mboya I.B., Jerkeman M., Stattin P., Wahlström J., Michaëlsson K., van Guelpen B. (2024). Body Mass Index and Risk of over 100 Cancer Forms and Subtypes in 4.1 Million Individuals in Sweden: The Obesity and Disease Development Sweden (ODDS) Pooled Cohort Study. Lancet Reg. Heal.—Eur..

[6] Powell-Wiley T.M., Poirier P., Burke L.E., Després J.P., Gordon-Larsen P., Lavie C.J., Lear S.A., Ndumele C.E., Neeland I.J., Sanders P. (2021). Obesity and Cardiovascular Disease A Scientific Statement From the American Heart Association. Circulation.

[7] Bhaskaran K., dos-Santos-Silva I., Leon D.A., Douglas I.J., Smeeth L. (2018). Association of BMI with Overall and Cause-Specific Mortality: A Population-Based Cohort Study of 3·6 Million Adults in the UK. Lancet Diabetes Endocrinol..

[8] Lanoye A., Brown K.L., LaRose J.G. (2017). The Transition into Young Adulthood: A Critical Period for Weight Control. Curr. Diab. Rep..

[9] Nelson M.C., Story M., Larson N.I., Neumark-Sztainer D., Lytle L.A. (2008). Emerging Adulthood and College-Aged Youth: An Overlooked Age for Weight-Related Behavior Change. Obesity.

[10] Bourke M., Brown D., Kwan M.Y.W. (2025). Lifestyle Behavior Patterns During the Transition From Adolescence to Emerging Adulthood: Associations with Mental Health and Wellbeing. Emerg. Adulthood.

[11] Tao Y., Wall M., Larson N., Neumark-Sztainer D., Winpenny E.M. (2024). Changes in Diet Quality across Life Transitions from Adolescence to Early Adulthood: A Latent Growth Analysis. Am. J. Clin. Nutr..

[12] Guo J.W., Ning H., Allen N.B., Perak A.M., Walker J.M., Pettee Gabriel K., Lloyd-Jones D.M. (2025). Cardiovascular Health Changes in Young Adults and Risk of Later-Life Cardiovascular Disease. JAMA Netw. Open.

[13] Nguyen M., Jarvis S.E., Tinajero M.G., Yu J., Chiavaroli L., Mejia S.B., Khan T.A., Tobias D.K., Willett W.C., Hu F.B. (2023). Sugar-Sweetened Beverage Consumption and Weight Gain in Children and Adults: A Systematic Review and Meta-Analysis of Prospective Cohort Studies and Randomized Controlled Trials. Am. J. Clin. Nutr..

[14] Malik V.S., Pan A., Willett W.C., Hu F.B. (2013). Sugar-Sweetened Beverages and Weight Gain in Children and Adults: A Systematic Review and Meta-Analysis. Am. J. Clin. Nutr..

[15] Dakanalis A., Mentzelou M., Papadopoulou S.K., Papandreou D., Spanoudaki M., Vasios G.K., Pavlidou E., Mantzorou M., Giaginis C. (2023). The Association of Emotional Eating with Overweight/Obesity, Depression, Anxiety/Stress, and Dietary Patterns: A Review of the Current Clinical Evidence. Nutrients.

[16] Vasileiou V., Abbott S. (2023). Emotional Eating among Adults with Healthy Weight, Overweight and Obesity: A Systematic Review and Meta-Analysis. J. Hum. Nutr. Diet..

[17] Ma X., Chen Q., Pu Y., Guo M., Jiang Z., Huang W., Long Y., Xu Y. (2020). Skipping Breakfast Is Associated with Overweight and Obesity: A Systematic Review and Meta-Analysis. Obes. Res. Clin. Pract..

[18] Direksunthorn T. (2025). Sleep and Cardiometabolic Health: A Narrative Review of Epidemiological Evidence, Mechanisms, and Interventions. Int. J. Gen. Med..

[19] Coven S., Jelic S., St-Onge M.-P. (2026). Fluctuations in Sleep Duration and Timing and Cardiometabolic Risk. Arterioscler. Thromb. Vasc. Biol..

[20] Yang W., Wu Y., Chen Y., Chen S., Gao X., Wu S., Sun L. (2024). Different Levels of Physical Activity and Risk of Developing Type 2 Diabetes among Adults with Prediabetes: A Population-Based Cohort Study. Nutr. J..

[21] Sulis S., Falbová D., Hozáková A., Vorobeľová L. (2025). Sex-Specific Interrelationships of Sleeping and Nutritional Habits with Somatic Health Indicators in Young Adults. Bratisl. Med. J..

[22] Falbová D., Sulis S., Oravská P., Hozaková A., Švábová P., Beňuš R., Vorobeľová L. (2025). The Prevalence of Normal Weight Obesity in Slovak Young Adults and Its Relationship with Body Composition and Lifestyle Habits. Bratisl. Med. J..

[23] Popa S., Moţa M., Popa A., Moţa E., Serafinceanu C., Guja C., Catrinoiu D., Hâncu N., Lichiardopol R., Bala C. (2016). Prevalence of Overweight/Obesity, Abdominal Obesity and Metabolic Syndrome and Atypical Cardiometabolic Phenotypes in the Adult Romanian Population: PREDATORR Study. J. Endocrinol. Investig..

[24] Roman G., Bala C., Creteanu G., Graur M., Morosanu M., Amorin P., Pircalaboiu L., Radulian G., Timar R., Achimas Cadariu A. (2015). Obesity and Health-Related Lifestyle Factors in the General Population in Romania: A Cross Sectional Study. Acta Endocrinol..

[25] Ispas A.G., Forray A.I., Lacurezeanu A., Petreuș D., Gavrilaș L.I., Cherecheș R.M. (2025). Talking About Weight with Children: Associations with Parental Stigma, Bias, Attitudes, and Child Weight Status. Nutrients.

[26] Roșioară A.I., Năsui B.A., Ciuciuc N., Sîrbu D.M., Curșeu D., Vesa Ș.C., Popescu C.A., Bleza A., Popa M. (2025). Beyond BMI: Exploring Adolescent Lifestyle and Health Behaviours in Transylvania, Romania. Nutrients.

[27] NIS Rezultate Definitive RPL 2021—Recensamantul Populatiei Si Locuintelor. https://www.recensamantromania.ro/rezultate-rpl-2021/rezultate-definitive/.

[28] Bull F.C., Al-Ansari S.S., Biddle S., Borodulin K., Buman M.P., Cardon G., Carty C., Chaput J.P., Chastin S., Chou R. (2020). World Health Organization 2020 Guidelines on Physical Activity and Sedentary Behaviour. Br. J. Sports Med..

[29] Hirshkowitz M., Whiton K., Albert S.M., Alessi C., Bruni O., DonCarlos L., Hazen N., Herman J., Adams Hillard P.J., Katz E.S. (2015). National Sleep Foundation’s Updated Sleep Duration Recommendations: Final Report. Sleep Health.

[30] Wicherski J., Schlesinger S., Fischer F. (2021). Association between Breakfast Skipping and Body Weight—A Systematic Review and Meta-Analysis of Observational Longitudinal Studies. Nutrients.

[31] Yanovski S.Z., Marcus M.D., Wadden T.A., Walsh B.T. (2015). The Questionnaire on Eating and Weight Patterns-5: An Updated Screening Instrument for Binge Eating Disorder. Int. J. Eat. Disord..

[32] Fung T.T., Chiuve S.E., McCullough M.L., Rexrode K.M., Logroscino G., Hu F.B. (2008). Adherence to a DASH-Style Diet and Risk of Coronary Heart Disease and Stroke in Women. Arch. Intern. Med..

[33] Shams-White M.M., Pannucci T.R.E., Lerman J.L., Herrick K.A., Zimmer M., Meyers Mathieu K., Stoody E.E., Reedy J. (2023). Healthy Eating Index-2020: Review and Update Process to Reflect the Dietary Guidelines for Americans, 2020–2025. J. Acad. Nutr. Diet..

[34] Trichopoulou A., Costacou T., Bamia C., Trichopoulos D. (2003). Adherence to a Mediterranean Diet and Survival in a Greek Population. N. Engl. J. Med..

[35] Rockström J., Thilsted S.H., Willett W.C., Gordon L.J., Herrero M., Hicks C.C., Mason-D’Croz D., Rao N., Springmann M., Wright E.C. (2025). The EAT–Lancet Commission on Healthy, Sustainable, and Just Food Systems. Lancet.

[36] Mozaffarian D., Hao T., Rimm E.B., Willett W.C., Hu F.B. (2011). Changes in Diet and Lifestyle and Long-Term Weight Gain in Women and Men. N. Engl. J. Med..

[37] Ramel A., Nwaru B.I., Lamberg-Allardt C., Thorisdottir B., Bärebring L., Söderlund F., Arnesen E.K., Dierkes J., Åkesson A. (2023). White Meat Consumption and Risk of Cardiovascular Disease and Type 2 Diabetes: A Systematic Review and Meta-Analysis. Food Nutr. Res..

[38] Formisano E., de Cassya Lopes Neri L., Caffa I., Borgarelli C., Ferrando M.R., Proietti E., Turrini F., Martini D., Angelino D., Tagliabue A. (2025). Effect of Egg Consumption on Health Outcomes: An Updated Umbrella Review of Systematic Reviews and Meta-Analysis of Observational and Intervention Studies. Nutr. Metab. Cardiovasc. Dis..

[39] Suárez E., Pérez C.M., Rivera R., Martínez M.N. (2017). Regression Models in a Cross-Sectional Study. Applications of Regression Models in Epidemiology.

[40] Segun A., Zhang B., Mary A.M., Kibenja D., Ma J., Said S., Adeniyi I., Barrow L.F. (2024). Exploring the Relationship between Dietary Patterns and Obesity among Nigerian Adults: A Cross-Sectional Study. BMC Public Health.

[41] Zhang Y., Li Y., Peila R., Wang T., Xue X., Kaplan R.C., Dannenberg A.J., Qi Q., Rohan T.E. (2024). Associations of Lifestyle and Genetic Risks with Obesity and Related Chronic Diseases in the UK Biobank: A Prospective Cohort Study. Am. J. Clin. Nutr..

[42] Juvanhol L.L., Lana R.M., Cabrelli R., Bastos L.S., Nobre A.A., Rotenberg L., Griep R.H. (2016). Factors Associated with Overweight: Are the Conclusions Influenced by Choice of the Regression Method?. BMC Public Health.

[43] Mititelu M., Popovici V., Neacșu S.M., Musuc A.M., Busnatu Ș.S., Oprea E., Boroghină S.C., Mihai A., Streba C.T., Lupuliasa D. (2024). Assessment of Dietary and Lifestyle Quality among the Romanian Population in the Post-Pandemic Period. Healthcare.

[44] Romero-Corral A., Somers V.K., Sierra-Johnson J., Korenfeld Y., Boarin S., Korinek J., Jensen M.D., Parati G., Lopez-Jimenez F. (2010). Normal Weight Obesity: A Risk Factor for Cardiometabolic Dysregulation and Cardiovascular Mortality. Eur. Heart J..

[45] Kaczmarek M. (2025). Normal Weight Obesity in Adolescents: Patterns and Associated Factors. Front. Nutr..

[46] Roman G., Rusu A., Graur M., Creteanu G., Morosanu M., Radulian G., Amorin P., Timar R., Pircalaboiu L., Bala C. (2019). Dietary Patterns and Their Association with Obesity: A Cross-Sectional Study. Acta Endocrinol..

[47] Lotrean L.M., Stan O., Lencu C., Laza V. (2018). Patrones Dietéticos, Actividad Física, Índice de Masa Corporal, Conductas Relacionadas Con El Peso y Su Interrelación Entre Estudiantes Universitarios Rumanos Entre 2003 y 2016. Nutr. Hosp..

[48] Rada C. (2016). Body Mass Index and Eating Habits in Young Adults from Romania. Int. J. Med. Res. Health Sci..

[49] Serban D.M., Ursoniu S., Moleriu R.D., Banu A.M., Serban C.L. (2024). Mindful Eating, Nutrition Knowledge, and Weight Status among Medical Students: Implications for Health and Counseling Practices. Nutrients.

[50] Mocanu V. (2013). Dieting Attitudes among College Students in Romania. Endocr. Abstr..

[51] Pescari D., Borlea A., Paul C., Mihuta S.M., Mozos I., Stoian D. (2025). Dietary Patterns and Behavioral Factors Among Adults with Obesity and Overweight in Western Romania: A Retrospective Observational Study. Timis. Med. J..

[52] Pescari D., Mihuta M.S., Bena A., Stoian D. (2024). Comparative Analysis of Dietary Habits and Obesity Prediction: Body Mass Index versus Body Fat Percentage Classification Using Bioelectrical Impedance Analysis. Nutrients.

[53] Barbu C.G., Teleman M.D., Albu A.I., Sirbu A.E., Martin S.C., Bancescu A., Fica S.V. (2015). Obesity and Eating Behaviors in School Children and Adolescents -Data from a Cross Sectional Study from Bucharest, Romania. BMC Public Health.

[54] Nasui B.A., Ungur R.A., Nasui G.A., Popescu C.A., Hofer A.M., Dersidan S., Popa M., Silaghi H., Silaghi C.A. (2023). Adolescents’ Lifestyle Determinants in Relation to Their Nutritional Status during COVID-19 Pandemic Distance Learning in the North-Western Part of Romania—A Cross-Sectional Study. Children.

[55] Ispas A.G., Forray A.I., Lacurezeanu A., Petreuș D., Gavrilaș L.I., Cherecheș R.M. (2025). Eating Disorder Risk Among Adolescents: The Influence of Dietary Patterns, Physical Activity, and BMI. Nutrients.

[56] Cappuccio F.P., Taggart F.M., Kandala N.B., Currie A., Peile E., Stranges S., Miller M.A. (2008). Meta-Analysis of Short Sleep Duration and Obesity in Children and Adults. Sleep.

[57] Wu Y., Zhai L., Zhang D. (2014). Sleep Duration and Obesity among Adults: A Meta-Analysis of Prospective Studies. Sleep Med..

[58] Palacios C., Raynor H.A., Anderson C.A., Andres A., Orlet Fisher J., Giovannucci E., Hoelscher D.M., Gardner C.D., Blue Bird Jernigan V., Odoms-Young A. (2024). Frequency of Meals and/or Snacking and Growth, Body Composition, and Risk of Obesity: A Systematic Review.

[59] Cowan A.E., Higgins K.A., Fisher J.O., Tripicchio G.L., Mattes R.D., Zou P., Bailey R.L. (2020). Examination of Different Definitions of Snacking Frequency and Associations with Weight Status among U.S. Adults. PLoS ONE.

[60] Marques A., Peralta M., Naia A., Loureiro N., De Matos M.G. (2018). Prevalence of Adult Overweight and Obesity in 20 European Countries, 2014. Eur. J. Public Health.

[61] Berghöfer A., Pischon T., Reinhold T., Apovian C.M., Sharma A.M., Willich S.N. (2008). Obesity Prevalence from a European Perspective: A Systematic Review. BMC Public Health.

[62] Grajek M., Krupa-Kotara K., Białek-Dratwa A., Staśkiewicz W., Rozmiarek M., Misterska E., Sas-Nowosielski K. (2022). Prevalence of Emotional Eating in Groups of Students with Varied Diets and Physical Activity in Poland. Nutrients.

[63] Sierpiński R., Jankowski M., Raciborski F. (2025). Differences in Lifestyle-Related Behaviors Among Healthy Weight, Overweight, and Obese Groups: A Secondary Analysis of Data on 4714 Adults in Poland. Nutrients.

[64] Kunzova M., Neto G.A.M., Infante-Garcia M.M., Nieto-Martinez R., González-Rivas J.P. (2022). Risk Factors Associated with the Consumption of Sugar-Sweetened Beverages among Czech Adults: The Kardiovize Study. Nutrients.

[65] Fuente González C.E., Chávez-Servín J.L., De La Torre-Carbot K., Ronquillo González D., Aguilera Barreiro M.D.L.Á., Ojeda Navarro L.R. (2022). Relationship between Emotional Eating, Consumption of Hyperpalatable Energy-Dense Foods, and Indicators of Nutritional Status: A Systematic Review. J. Obes..

[66] Ramalho S.M., Conceição E., Tavares A.C., Freitas A.L., Machado B.C., Gonçalves S. (2023). Loss of Control over Eating, Inhibitory Control, and Reward Sensitivity in Children and Adolescents: A Systematic Review. Nutrients.

[67] Jayedi A., Soltani S., Emadi A., Zargar M.S., Najafi A. (2024). Aerobic Exercise and Weight Loss in Adults: A Systematic Review and Dose-Response Meta-Analysis. JAMA Netw. Open.

[68] Klein S., Allison D.B., Heymsfield S.B., Kelley D.E., Leibel R.L., Nonas C., Kahn R. (2007). Waist Circumference and Cardiometabolic Risk: A Consensus Statement from Shaping America’s Health: Association for Weight Management and Obesity Prevention; NAASO, The Obesity Society; the American Society for Nutrition; and the American Diabetes Association. Am. J. Clin. Nutr..

[69] Lim S., Wyker B., Bartley K., Eisenhower D. (2015). Measurement Error of Self-Reported Physical Activity Levels in New York City: Assessment and Correction. Am. J. Epidemiol..

[70] Falbová D., Beňuš R., Vorobelová L. (2023). Association between Smoking Status and Body Composition Parameters in a Young Adult Population. Anthropol. Rev..

[71] Negrea M.O., Negrea G.O., Săndulescu G., Neamtu B., Solomon A., Popa M.L., Stoia O., Domnariu C.D., Teodoru M. (2024). Assessing Lifestyle Patterns and Their Influence on Weight Status in Students from a High School in Sibiu, Romania: An Adaptation of ISCOLE Questionnaires and the Child Feeding Questionnaire. Nutrients.

[72] Lupu C.E., Scafa-Udriște A., Matei R.S., Licu M., Stanciu T.I., Stanciu G., Hashemi F., Mihai A., Lupu S., Ene R. (2025). Adolescent Nutritional Patterns and Health Behaviors in Romania: A Cross-Sectional Analysis. Nutrents.

