# Supplementary material for: Lifestyle and Dietary Behaviors Are Associated with Body Mass Index in Romanian Young Adults
Source: Nutrients. 2026 May 21;18(10):1644. doi: 10.3390/nu18101644 (PMC13209717; doi:10.3390/nu18101644)
Supplement: Supplementary file 1 [file nutrients-18-01644-s001.zip › nutrients-4316921-supplementary.pdf]

Supplement

Study Questionnaire (English Translation)

Original language: Romanian. Administered via Google Forms.

Part 1: General Information

1. Sex: *

○ Female
○ Male

2. Age: *

[Free-text response]

3. Marital status: *

○ Married or in a stable relationship
○ Unmarried

4. Citizenship: *

○ Romanian
○ Other: ___________

5. Place of residence: *

○ Urban
○ Rural

6. In which region of the country do you live? *

○ Nord-Est (Counties: Bacău, Botoșani, Iași, Neamț, Suceava, Vaslui)
○ Sud-Est (Counties: Brăila, Buzău, Constanța, Galați, Tulcea, Vrancea)
○ Sud-Muntenia (Counties: Argeș, Călărași, Dâmbovița, Giurgiu, Ialomița, Prahova, Teleorman)
○ Sud-Vest Oltenia (Counties: Dolj, Gorj, Mehedinți, Olt, Vâlcea)
○ Vest (Counties: Arad, Caraș-Severin, Hunedoara, Timiș)
○ Nord-Vest (Counties: Bihor, Bistrița-Năsăud, Cluj, Maramureș, Satu-Mare, Sălaj)
○ Centru (Counties: Alba, Brașov, Covasna, Harghita, Mureș, Sibiu)
○ București–Ilfov
○ Diaspora

7. Level of education: *

○ Middle school
○ Secondary education (high school, vocational school)
○ University education

8. How would you describe the nature of your professional activity? *

○ I spend most of my time at a desk, sitting (e.g., student)
○ I spend most of my time standing or walking (e.g., cashier, hairdresser, etc.)
○ My work involves intense physical activity, including handling heavy objects (e.g., construction worker, agriculture, etc.)

9. What is the net monthly income of your household? *

- ○ Below 2,000 lei
- ○ Between 2,000 and 4,000 lei
- ○ Between 4,000 and 6,000 lei
- ○ Between 6,000 and 8,000 lei
- ○ Between 8,000 and 10,000 lei
- ○ Over 10,000 lei

10. Height (cm): *

[Free-text response]

11. Weight (kg): *

[Free-text response]

12. Have you been diagnosed with any cardiometabolic condition (type 2 diabetes mellitus, arterial hypertension, atherosclerosis, dyslipidaemia, gout, etc.)? *

- ○ No
- ○ Yes

13. With which cardiometabolic condition have you been diagnosed?

Displayed only if Q12 = Yes. Multiple selections allowed.

- ☐ Type 2 diabetes mellitus
- ☐ Arterial hypertension
- ☐ Atherosclerosis
- ☐ Dyslipidaemia
- ☐ Gout
- ☐ Other: ____________

Part 2: Lifestyle

1. What is your level of physical activity (PA)? Moderate physical activity: increases heart rate and breathing, but you can still talk (e.g., brisk walking, cycling). Vigorous physical activity: significantly increases pulse and breathing, and talking becomes difficult (e.g., running, intense sports). *

- ○ I do not engage in PA at all
- ○ <1–74 minutes/week of vigorous PA or 1–149 minutes/week of moderate PA
- ○ ≥75 minutes/week (10 minutes/day) of sustained/vigorous PA or ≥150 minutes/week (30 minutes/day) of moderate PA

2. Are you a smoker? *

- ○ Yes
- ○ No
- ○ I was a smoker, but I quit less than 6 months ago
- ○ I was a smoker, but I quit more than 6 months ago

3. Do you experience sleep problems (nocturnal awakenings or difficulty falling asleep) at least 3 times per week? *

- ○ Yes
- ○ No

4. If you answered yes, what type of sleep problem do you experience most frequently?

Displayed only if Q3 = Yes. Multiple selections allowed.

- ☐ Difficulty falling asleep
- ☐ Frequent awakenings during the night
- ☐ Restless or poor-quality sleep
- ☐ Waking up too early in the morning and inability to fall back asleep
- ☐ Other problems

5. How long do you sleep per night? *

- ○ <3 hours
- ○ 3–5 hours
- ○ 6–8 hours
- ○ >8 hours

6. How many servings of alcohol do you consume on average per week, considering that one serving equals: 330 ml regular beer (≈5% alcohol) = 250 ml cider/liqueur in a 350 ml glass (≈7% alcohol) = 150 ml table wine (≈12% alcohol) = 45 ml spirits such as tequila, vodka, whiskey, etc. (≈40% alcohol). *

- ○ None
- ○ <7 servings/week
- ○ Between 7 and 14 servings/week
- ○ >14 servings/week

7. Have you experienced any of the following situations in the last 6 months? *

- ☐ Depression
- ☐ Stress
- ☐ Sudden mood changes

- ☐ Fatigue
- ☐ Social isolation
- ☐ Feelings of anger
- ☐ None
- ☐ Other: ___________

8. In the past week, what was your perception of your stress level in the following situations? *

Matrix: rows = “At the workplace”, “At home”; columns = “Never”, “Sometimes”, “Often”, “Always”.

Part 3: Nutritional Assessment

1. What dietary pattern do you currently follow? *

○ Omnivore (You consume all types of food, including meat, dairy, eggs, fruit, vegetables, and cereals)

○ Pescatarian (You consume primarily plant-based foods, but also include fish or seafood in your diet, without other types of meat)

○ Vegetarian (You consume only plant-based foods, completely eliminating meat, fish, and seafood. Your diet may include eggs and dairy)

○ Vegan (You consume exclusively plant-based foods, completely avoiding any animal-derived products, including eggs, dairy, honey, or other derivatives)

○ Raw vegan (You consume exclusively plant-based foods in their raw state or thermally processed at temperatures below 40–48°C, to preserve natural enzymes and unaltered nutrients)

2. Water consumption: *

○ <1 L/day

○ 1–2 L/day

○ >2 L/day

3. How many main meals do you have, on average, per day? *

[Free-text response]

4. How many snacks do you have, on average, per day? *

[Free-text response]

5. How many times per week do you have: *

Matrix: rows = "Breakfast", "Lunch", "Dinner"; columns = 1, 2, 3, 4, 5, 6, 7.

6. Do you read food labels before purchasing? *

○ Yes

○ No

○ Sometimes or only for some products

7. Please indicate the frequency with which you usually: *

Matrix: rows = "Cook at home", "Order food from a restaurant/fast food", "Eat alone", "Eat compulsively (repetitive, uncontrollable behaviour)"; columns = "Less than once per week", "1–3 times/week", "4–6 times/week", "Once a day or more".

8. In a typical week, how often do you eat/drink the following foods/beverages? *

Matrix: rows = "Vegetables", "Fruit", "Leafy green vegetables", "Red and orange fruit & vegetables", "Milk", "Dairy products", "Eggs", "Fish", "Seafood", "White meat", "Red meat", "Processed meat (cold cuts)", "Bread, pasta, or cereals", "Wholegrain bread, pasta, or cereals", "Legumes (beans, peas, chickpeas, soya)", "Nuts and seeds (walnuts, almonds, hazelnuts)", "Deep-fried foods (schnitzel, breaded cheese, meatballs, french fries, etc.)", "Sweets", "Pastry", "Coffee", "Tea", "Carbonated beverages (Coca-Cola, Pepsi, Sprite, etc.)", "Natural juice"; columns = "Less than once per week", "1–3 times/week", "4–6 times/week", "Once a day or more".

Article

Supplementary material of the article Lifestyle and Dietary Behaviors Are Associated with Body Mass Index in Romanian Young Adults

Diana Crișan[1], Oleg Frumuzachi[1,2,*], Denisia Pașca[2,3], Laura Gavrilaş[2,3,*], and Gianina Crișan[1]

[1] Department of Pharmaceutical Botany, Faculty of Pharmacy, "Iuliu Hațieganu" University of Medicine and Pharmacy, 23 Gheorghe Marinescu Street, 400337 Cluj-Napoca, Romania; diana.suciu.crisan@elearn.umfcluj.ro (D.C.), oleg.frumuzachi@elearn.umfcluj.ro (O.F.), gcrisan@umfcluj.ro (G.C.);

[2] The Romanian Dietitians Association, 6 Dionisie Roman street, 400595 Cluj-Napoca, Romania;

[3] Department 2, Faculty of Nursing and Health Sciences, "Iuliu Hațieganu" University of Medicine and Pharmacy, 23 Gheorghe Marinescu Street, 400337 Cluj-Napoca, Romania; denisia.pasca@umfcluj.ro (D.P.), laura.gavrilas@umfcluj.ro (L.G.);

* Correspondence: oleg.frumuzachi@elearn.umfcluj.ro (O.F.), laura.gavrilas@umfcluj.ro (L.G.).

Abstract

Background/Objectives: Overweight and obesity are increasing globally. However, structured contemporary data on lifestyle behaviors and adiposity in Romanian young adults remain limited. Therefore, this study aimed to describe dietary and lifestyle habits, BMI, and overweight/obesity prevalence in Romanian adults aged 18–30 years and to examine associations between these variables. **Methods:** This cross-sectional online questionnaire study included 1202 young Romanian adults. BMI was calculated from self-reported height and weight and analyzed continuously, as well as for overweight/obesity (BMI ≥25 kg/m^2). Pre-specified exposures were compulsive eating, soft-drink intake, breakfast frequency, physical activity, and sleep duration. Multivariable linear regression with heteroscedasticity-consistent standard errors was used for BMI, and modified Poisson regression with robust variance was used for overweight/obesity. Composite dietary score, sex-interaction, and sensitivity analyses were also performed. **Results:** Mean age was 23.2 ± 3.3 years, mean BMI was 23.8 ± 4.2 kg/m^2, and 32.4% of participants had overweight/obesity. Men had higher BMI and a higher prevalence of overweight/obesity than women. Compulsive eating and soft-drink intake showed dose-dependent associations with higher BMI and higher overweight/obesity prevalence. Short sleep duration (≤5 h/night) and daily breakfast consumption were associated with a higher and, respectively, lower prevalence of overweight/obesity. Physical activity showed no independent association after full adjustment, although this finding may be influenced by the use of a single self-reported item. Composite-score analyses supported the main findings. **Conclusions:** In Romanian young adults, compulsive eating and soft-drink intake were the most consistent behavioral correlates of adiposity, while breakfast regularity and short sleep showed threshold-type associations with overweight/obesity. These findings may inform the design of multicomponent prevention strategies, although longitudinal confirmation is needed.

Table S1. Food-consumption profile, overall and by sex.

Food	Frequency	Overall	Female	Male	P (overall)	P (level)
Vegetables		n = 1202	n = 563	n = 639	<0.001	
	<1/wk	52 (4.3%)	20 (3.6%)	32 (5.0%)		0.273
	1-3/wk	338 (28.1%)	123 (21.8%)	215 (33.6%)		<0.001
	4-6/wk	400 (33.3%)	172 (30.6%)	228 (35.7%)		0.068
	≥1/day	412 (34.3%)	248 (44.0%)	164 (25.7%)		<0.001
Fruit		n = 1202	n = 563	n = 639	<0.001	
	<1/wk	110 (9.2%)	37 (6.6%)	73 (11.4%)		0.005
	1-3/wk	475 (39.5%)	201 (35.7%)	274 (42.9%)		0.013
	4-6/wk	322 (26.8%)	149 (26.5%)	173 (27.1%)		0.863
	≥1/day	295 (24.5%)	176 (31.3%)	119 (18.6%)		<0.001
Leafy greens		n = 1202	n = 563	n = 639	0.112	
	<1/wk	191 (15.9%)	82 (14.6%)	109 (17.1%)		0.271
	1-3/wk	566 (47.1%)	271 (48.1%)	295 (46.2%)		0.532
	4-6/wk	267 (22.2%)	115 (20.4%)	152 (23.8%)		0.184
	≥1/day	178 (14.8%)	95 (16.9%)	83 (13.0%)		0.070
Red/orange fruit & veg		n = 1202	n = 563	n = 639	0.002	
	<1/wk	140 (11.6%)	52 (9.2%)	88 (13.8%)		0.018
	1-3/wk	525 (43.7%)	234 (41.6%)	291 (45.5%)		0.184
	4-6/wk	352 (29.3%)	171 (30.4%)	181 (28.3%)		0.475
	≥1/day	185 (15.4%)	106 (18.8%)	79 (12.4%)		0.003
Milk		n = 1202	n = 563	n = 639	<0.001	
	<1/wk	312 (26.0%)	152 (27.0%)	160 (25.0%)		0.479
	1-3/wk	356 (29.6%)	140 (24.9%)	216 (33.8%)		<0.001
	4-6/wk	279 (23.2%)	125 (22.2%)	154 (24.1%)		0.478
	≥1/day	255 (21.2%)	146 (25.9%)	109 (17.1%)		<0.001
Dairy products		n = 1202	n = 563	n = 639	0.002	
	<1/wk	116 (9.7%)	56 (9.9%)	60 (9.4%)		0.819
	1-3/wk	384 (31.9%)	163 (29.0%)	221 (34.6%)		0.043
	4-6/wk	440 (36.6%)	195 (34.6%)	245 (38.3%)		0.204
	≥1/day	262 (21.8%)	149 (26.5%)	113 (17.7%)		<0.001
Eggs		n = 1202	n = 563	n = 639	0.202	
	<1/wk	145 (12.1%)	75 (13.3%)	70 (11.0%)		0.243
	1-3/wk	553 (46.0%)	254 (45.1%)	299 (46.8%)		0.600
	4-6/wk	349 (29.0%)	171 (30.4%)	178 (27.9%)		0.370
	≥1/day	155 (12.9%)	63 (11.2%)	92 (14.4%)		0.117
Fish		n = 1202	n = 563	n = 639	0.064	
	<1/wk	735 (61.1%)	337 (59.9%)	398 (62.3%)		0.422
	1-3/wk	372 (30.9%)	191 (33.9%)	181 (28.3%)		0.042
	4-6/wk	70 (5.8%)	25 (4.4%)	45 (7.0%)		0.072

Food	Frequency	Overall	Female	Male	P (overall)	P (level)
	≥1/day	25 (2.1%)	10 (1.8%)	15 (2.3%)		0.624
Seafood		n = 1202	n = 563	n = 639	0.007	
	<1/wk	1023 (85.1%)	498 (88.5%)	525 (82.2%)		0.003
	1-3/wk	140 (11.6%)	54 (9.6%)	86 (13.5%)		0.046
	4-6/wk	29 (2.4%)	7 (1.2%)	22 (3.4%)		0.022
	≥1/day	10 (0.8%)	4 (0.7%)	6 (0.9%)		0.758
White meat		n = 1202	n = 563	n = 639	0.769	
	<1/wk	166 (13.8%)	79 (14.0%)	87 (13.6%)		0.900
	1-3/wk	414 (34.4%)	190 (33.7%)	224 (35.1%)		0.678
	4-6/wk	463 (38.5%)	224 (39.8%)	239 (37.4%)		0.430
	≥1/day	159 (13.2%)	70 (12.4%)	89 (13.9%)		0.498
Red meat		n = 1202	n = 563	n = 639	<0.001	
	<1/wk	375 (31.2%)	245 (43.5%)	130 (20.3%)		<0.001
	1-3/wk	514 (42.8%)	233 (41.4%)	281 (44.0%)		0.397
	4-6/wk	256 (21.3%)	70 (12.4%)	186 (29.1%)		<0.001
	≥1/day	57 (4.7%)	15 (2.7%)	42 (6.6%)		0.002
Processed meat		n = 1202	n = 563	n = 639	<0.001	
	<1/wk	447 (37.2%)	251 (44.6%)	196 (30.7%)		<0.001
	1-3/wk	441 (36.7%)	185 (32.9%)	256 (40.1%)		0.012
	4-6/wk	248 (20.6%)	102 (18.1%)	146 (22.8%)		0.051
	≥1/day	66 (5.5%)	25 (4.4%)	41 (6.4%)		0.170
Bread/pasta/cereals		n = 1202	n = 563	n = 639	0.508	
	<1/wk	152 (12.6%)	78 (13.9%)	74 (11.6%)		0.273
	1-3/wk	361 (30.0%)	162 (28.8%)	199 (31.1%)		0.406
	4-6/wk	393 (32.7%)	189 (33.6%)	204 (31.9%)		0.586
	≥1/day	296 (24.6%)	134 (23.8%)	162 (25.4%)		0.578
Whole grains		n = 1202	n = 563	n = 639	0.509	
	<1/wk	338 (28.1%)	154 (27.4%)	184 (28.8%)		0.624
	1-3/wk	370 (30.8%)	165 (29.3%)	205 (32.1%)		0.329
	4-6/wk	278 (23.1%)	136 (24.2%)	142 (22.2%)		0.468
	≥1/day	216 (18.0%)	108 (19.2%)	108 (16.9%)		0.341
Legumes		n = 1202	n = 563	n = 639	0.228	
	<1/wk	432 (35.9%)	197 (35.0%)	235 (36.8%)		0.560
	1-3/wk	507 (42.2%)	236 (41.9%)	271 (42.4%)		0.909
	4-6/wk	204 (17.0%)	107 (19.0%)	97 (15.2%)		0.092
	≥1/day	59 (4.9%)	23 (4.1%)	36 (5.6%)		0.269
Nuts/seeds		n = 1202	n = 563	n = 639	0.017	
	<1/wk	456 (37.9%)	198 (35.2%)	258 (40.4%)		0.072
	1-3/wk	476 (39.6%)	216 (38.4%)	260 (40.7%)		0.446
	4-6/wk	180 (15.0%)	100 (17.8%)	80 (12.5%)		0.014
	≥1/day	90 (7.5%)	49 (8.7%)	41 (6.4%)		0.163

Food	Frequency	Overall	Female	Male	P (overall)	P (level)
Fried foods		n = 1202	n = 563	n = 639	<0.001	
	<1/wk	453 (37.7%)	260 (46.2%)	193 (30.2%)		<0.001
	1-3/wk	472 (39.3%)	203 (36.1%)	269 (42.1%)		0.037
	4-6/wk	229 (19.1%)	80 (14.2%)	149 (23.3%)		<0.001
	≥1/day	48 (4.0%)	20 (3.6%)	28 (4.4%)		0.558
Sweets		n = 1202	n = 563	n = 639	0.002	
	<1/wk	250 (20.8%)	96 (17.1%)	154 (24.1%)		0.003
	1-3/wk	444 (36.9%)	203 (36.1%)	241 (37.7%)		0.593
	4-6/wk	308 (25.6%)	153 (27.2%)	155 (24.3%)		0.275
	≥1/day	200 (16.6%)	111 (19.7%)	89 (13.9%)		0.009
Pastry		n = 1202	n = 563	n = 639	0.007	
	<1/wk	467 (38.9%)	241 (42.8%)	226 (35.4%)		0.010
	1-3/wk	495 (41.2%)	222 (39.4%)	273 (42.7%)		0.272
	4-6/wk	180 (15.0%)	68 (12.1%)	112 (17.5%)		0.010
	≥1/day	60 (5.0%)	32 (5.7%)	28 (4.4%)		0.367
Coffee		n = 1202	n = 563	n = 639	0.001	
	<1/wk	328 (27.3%)	152 (27.0%)	176 (27.5%)		0.883
	1-3/wk	207 (17.2%)	75 (13.3%)	132 (20.7%)		0.001
	4-6/wk	186 (15.5%)	84 (14.9%)	102 (16.0%)		0.675
	≥1/day	481 (40.0%)	252 (44.8%)	229 (35.8%)		0.002
Tea		n = 1202	n = 563	n = 639	<0.001	
	<1/wk	547 (45.5%)	212 (37.7%)	335 (52.4%)		<0.001
	1-3/wk	343 (28.5%)	155 (27.5%)	188 (29.4%)		0.509
	4-6/wk	161 (13.4%)	90 (16.0%)	71 (11.1%)		0.017
	≥1/day	151 (12.6%)	106 (18.8%)	45 (7.0%)		<0.001
Soft drinks		n = 1202	n = 563	n = 639	<0.001	
	<1/wk	606 (50.4%)	355 (63.1%)	251 (39.3%)		<0.001
	1-3/wk	323 (26.9%)	130 (23.1%)	193 (30.2%)		0.007
	4-6/wk	169 (14.1%)	50 (8.9%)	119 (18.6%)		<0.001
	≥1/day	104 (8.7%)	28 (5.0%)	76 (11.9%)		<0.001
Natural juice		n = 1202	n = 563	n = 639	<0.001	
	<1/wk	664 (55.2%)	348 (61.8%)	316 (49.5%)		<0.001
	1-3/wk	378 (31.4%)	161 (28.6%)	217 (34.0%)		0.053
	4-6/wk	117 (9.7%)	39 (6.9%)	78 (12.2%)		0.003
	≥1/day	43 (3.6%)	15 (2.7%)	28 (4.4%)		0.149

Table S2. Full regression results (Model 1 vs Model 2)

Table S2A. Full multivariable linear regression for BMI: Model 1 (age + sex) vs Model 2 (fully adjusted), all terms shown.

Term	Model 1 β (95% CI)	P (M1)	Model 2 β (95% CI)	P (M2)
PA: Insufficient (vs Inactive)	-0.46 (-1.52 to 0.59)	0.390	-0.41 (-1.46 to 0.65)	0.448
PA: Meeting guidelines (vs Inactive)	-0.25 (-1.32 to 0.83)	0.654	-0.20 (-1.27 to 0.87)	0.714
Sleep: ≤5 h (vs 6-8 h)	0.84 (-0.16 to 1.83)	0.098	0.79 (-0.22 to 1.80)	0.124
Sleep: >8 h (vs 6-8 h)	-0.04 (-0.64 to 0.56)	0.903	-0.02 (-0.63 to 0.59)	0.952
Soft drinks: 1-3/wk (vs <1/wk)	0.73 (0.20 to 1.26)	0.007	0.55 (0.01 to 1.09)	0.045
Soft drinks: 4-6/wk (vs <1/wk)	1.12 (0.34 to 1.90)	0.005	1.05 (0.25 to 1.84)	0.010
Soft drinks: ≥1/day (vs <1/wk)	1.48 (0.58 to 2.38)	0.001	1.29 (0.39 to 2.19)	0.005
Compulsive eating: 1-3/wk (vs <1/wk)	1.10 (0.57 to 1.64)	<0.001	1.15 (0.60 to 1.69)	<0.001
Compulsive eating: 4-6/wk (vs <1/wk)	2.63 (1.63 to 3.62)	<0.001	2.60 (1.59 to 3.61)	<0.001
Compulsive eating: ≥1/day (vs <1/wk)	2.88 (1.28 to 4.48)	<0.001	3.00 (1.38 to 4.62)	<0.001
Breakfast: 3-4/wk (vs ≤2/wk)	-0.00 (-0.72 to 0.71)	0.995	-0.02 (-0.76 to 0.72)	0.967
Breakfast: 5-6/wk (vs ≤2/wk)	-0.23 (-0.93 to 0.47)	0.522	-0.19 (-0.90 to 0.52)	0.599
Breakfast: Daily (vs ≤2/wk)	-0.38 (-0.98 to 0.23)	0.226	-0.28 (-0.92 to 0.36)	0.389
Age (per year)	0.25 (0.18 to 0.32)	<0.001	0.26 (0.18 to 0.34)	<0.001
Male (vs Female)	2.40 (1.91 to 2.88)	<0.001	2.25 (1.72 to 2.77)	<0.001
Current smoker (vs not)			0.73 (0.14 to 1.33)	0.016
Alcohol (ordinal, per step)			-0.23 (-0.60 to 0.14)	0.223
Rural (vs Urban)			0.32 (-0.30 to 0.94)	0.307
Education (ordinal, per step)			-0.16 (-0.74 to 0.41)	0.575
Income (ordinal, per step)			0.03 (-0.10 to 0.17)	0.648
Work stress (ordinal, per step)			-0.21 (-0.48 to 0.06)	0.128
Region: Center (vs North-West)			-0.50 (-1.10 to 0.10)	0.100
Region: Bucharest-Ilfov (vs North-West)			-0.65 (-1.41 to 0.11)	0.093
Region: North-East (vs North-West)			0.03 (-0.84 to 0.89)	0.954
Region: West (vs North-West)			-0.20 (-1.21 to 0.80)	0.690
Region: South-East (vs North-West)			-0.34 (-1.93 to 1.24)	0.671
Region: Diaspora (vs North-West)			-0.44 (-2.15 to 1.27)	0.614
Region: South-Muntenia (vs North-West)			-0.87 (-2.27 to 0.53)	0.224
Region: South-Vest Oltenia (vs North-West)			0.91 (-0.67 to 2.50)	0.259

Table S2B. Full PR models for overweight/obesity: Model 1 vs Model 2, all terms shown.

Term	Model 1 PR (95% CI)	P (M1)	Model 2 PR (95% CI)	P (M2)
PA: Insufficient (vs Inactive)	0.91 (0.71 to 1.17)	0.457	0.94 (0.73 to 1.20)	0.597
PA: Meeting guidelines (vs Inactive)	0.94 (0.73 to 1.21)	0.622	0.94 (0.73 to 1.21)	0.612
Sleep: ≤5 h (vs 6-8 h)	1.37 (1.09 to 1.72)	0.007	1.34 (1.05 to 1.69)	0.016
Sleep: >8 h (vs 6-8 h)	1.02 (0.80 to 1.31)	0.846	1.03 (0.81 to 1.31)	0.811
Soft drinks: 1-3/wk (vs <1/wk)	1.38 (1.14 to 1.68)	0.001	1.27 (1.04 to 1.55)	0.017
Soft drinks: 4-6/wk (vs <1/wk)	1.31 (1.04 to 1.66)	0.023	1.27 (1.01 to 1.60)	0.044

Term	Model 1 PR (95% CI)	P (M1)	Model 2 PR (95% CI)	P (M2)
Soft drinks: ≥1/day (vs <1/wk)	1.58 (1.24 to 2.00)	<0.001	1.42 (1.11 to 1.82)	0.005
Compulsive eating: 1-3/wk (vs <1/wk)	1.21 (1.01 to 1.45)	0.035	1.24 (1.03 to 1.48)	0.020
Compulsive eating: 4-6/wk (vs <1/wk)	1.77 (1.39 to 2.24)	<0.001	1.73 (1.36 to 2.19)	<0.001
Compulsive eating: ≥1/day (vs <1/wk)	1.75 (1.27 to 2.39)	<0.001	1.86 (1.35 to 2.57)	<0.001
Breakfast: 3-4/wk (vs ≤2/wk)	0.91 (0.73 to 1.12)	0.352	0.90 (0.73 to 1.11)	0.313
Breakfast: 5-6/wk (vs ≤2/wk)	0.90 (0.72 to 1.14)	0.382	0.89 (0.71 to 1.11)	0.297
Breakfast: Daily (vs ≤2/wk)	0.74 (0.60 to 0.90)	0.003	0.75 (0.61 to 0.92)	0.007
Age (per year)	1.08 (1.05 to 1.10)	<0.001	1.07 (1.04 to 1.09)	<0.001
Male (vs Female)	2.01 (1.66 to 2.43)	<0.001	1.91 (1.56 to 2.34)	<0.001
Current smoker (vs not)			1.32 (1.10 to 1.58)	0.002
Alcohol (ordinal, per step)			0.95 (0.84 to 1.06)	0.355
Rural (vs Urban)			1.16 (0.96 to 1.40)	0.136
Education (ordinal, per step)			0.94 (0.79 to 1.13)	0.521
Income (ordinal, per step)			1.07 (1.02 to 1.13)	0.004
Work stress (ordinal, per step)			0.91 (0.83 to 1.00)	0.052
Region: Center (vs North-West)			0.93 (0.75 to 1.15)	0.518
Region: Bucharest-Ilfov (vs North-West)			0.93 (0.67 to 1.31)	0.687
Region: North-East (vs North-West)			1.06 (0.77 to 1.46)	0.734
Region: West (vs North-West)			1.39 (1.03 to 1.87)	0.031
Region: South-East (vs North-West)			1.04 (0.62 to 1.72)	0.893
Region: Diaspora (vs North-West)			0.79 (0.49 to 1.27)	0.329
Region: South-Muntenia (vs North-West)			0.77 (0.36 to 1.67)	0.512
Region: South-Vest Oltenia (vs North-West)			1.41 (0.96 to 2.06)	0.080

Table S3. Full composite-score models (Model 3)

Table S3A. Full composite-score linear regression for BMI (Model 3, all terms).

Term	β (95% CI)	P
Healthy-food score	-0.11 (-0.54 to 0.32)	0.617
Unhealthy-food score	0.42 (-0.03 to 0.87)	0.065
Meal regularity score	-0.08 (-0.14 to -0.02)	0.008
Age (per year)	0.24 (0.16 to 0.32)	<0.001
Male (vs Female)	2.24 (1.74 to 2.75)	<0.001
Current smoker (vs not)	1.00 (0.40 to 1.59)	0.001
Alcohol (ordinal, per step)	-0.20 (-0.59 to 0.18)	0.292
Rural (vs Urban)	0.38 (-0.25 to 1.01)	0.234
Education (ordinal, per step)	-0.14 (-0.72 to 0.44)	0.639
Income (ordinal, per step)	0.03 (-0.11 to 0.17)	0.645
Work stress (ordinal, per step)	-0.09 (-0.37 to 0.19)	0.545
Region: Center (vs North-West)	-0.54 (-1.14 to 0.05)	0.075
Region: Bucharest-Ilfov (vs North-West)	-0.53 (-1.29 to 0.24)	0.176
Region: North-East (vs North-West)	0.07 (-0.81 to 0.96)	0.870
Region: West (vs North-West)	-0.04 (-1.09 to 1.01)	0.942
Region: South-East (vs North-West)	-0.32 (-1.91 to 1.28)	0.698
Region: Diaspora (vs North-West)	-0.29 (-1.90 to 1.33)	0.728
Region: South-Muntenia (vs North-West)	-1.33 (-2.75 to 0.10)	0.068
Region: South-Vest Oltenia (vs North-West)	0.78 (-0.95 to 2.52)	0.377

Table S3B. Full composite-score PR model for overweight/obesity (Model 3, all terms).

Term	PR (95% CI)	P
Healthy-food score	0.92 (0.79 to 1.06)	0.250
Unhealthy-food score	1.13 (0.99 to 1.29)	0.062
Meal regularity score	0.97 (0.95 to 0.98)	<0.001
Age (per year)	1.06 (1.04 to 1.09)	<0.001
Male (vs Female)	1.85 (1.52 to 2.25)	<0.001
Current smoker (vs not)	1.43 (1.20 to 1.70)	<0.001
Alcohol (ordinal, per step)	0.95 (0.85 to 1.07)	0.414
Rural (vs Urban)	1.16 (0.96 to 1.40)	0.131
Education (ordinal, per step)	0.97 (0.81 to 1.15)	0.699
Income (ordinal, per step)	1.07 (1.02 to 1.13)	0.004
Work stress (ordinal, per step)	0.94 (0.85 to 1.03)	0.186
Region: Center (vs North-West)	0.93 (0.76 to 1.15)	0.514
Region: Bucharest-Ilfov (vs North-West)	0.95 (0.68 to 1.31)	0.735
Region: North-East (vs North-West)	1.06 (0.77 to 1.46)	0.729
Region: West (vs North-West)	1.47 (1.08 to 1.99)	0.014
Region: South-East (vs North-West)	1.03 (0.61 to 1.74)	0.902
Region: Diaspora (vs North-West)	0.81 (0.52 to 1.28)	0.370

Term	PR (95% CI)	P
Region: South-Muntenia (vs North-West)	0.68 (0.32 to 1.44)	0.319
Region: South-Vest Oltenia (vs North-West)	1.35 (0.88 to 2.06)	0.166

Table S4. Sex × exposure interaction tests

Exposure	Δdf	F	P-interaction
Physical activity	2	1.89	0.152
Sleep duration	2	0.10	0.902
Soft drinks	3	0.88	0.450
Compulsive eating	3	1.59	0.189
Breakfast frequency	3	1.38	0.248

Table S5. Sensitivity analyses

Table S5A. Sensitivity analyses for BMI (main exposures).

Sensitivity	Term	Estimate (95% CI)	P
	PA: Insufficient (vs Inactive)	-0.41 (-1.46 to 0.65)	0.448
	PA: Meeting guidelines (vs Inactive)	-0.20 (-1.27 to 0.87)	0.714
	Sleep: ≤5 h (vs 6-8 h)	0.79 (-0.22 to 1.80)	0.124
	Sleep: >8 h (vs 6-8 h)	-0.02 (-0.63 to 0.59)	0.952
	Soft drinks: 1-3/wk (vs <1/wk)	0.55 (0.01 to 1.09)	0.045
	Soft drinks: 4-6/wk (vs <1/wk)	1.05 (0.25 to 1.84)	0.010
Main model	Soft drinks: ≥1/day (vs <1/wk)	1.29 (0.39 to 2.19)	0.005
	Compulsive eating: 1-3/wk (vs <1/wk)	1.15 (0.60 to 1.69)	<0.001
	Compulsive eating: 4-6/wk (vs <1/wk)	2.60 (1.59 to 3.61)	<0.001
	Compulsive eating: ≥1/day (vs <1/wk)	3.00 (1.38 to 4.62)	<0.001
	Breakfast: 3-4/wk (vs ≤2/wk)	-0.02 (-0.76 to 0.72)	0.967
	Breakfast: 5-6/wk (vs ≤2/wk)	-0.19 (-0.90 to 0.52)	0.599
	Breakfast: Daily (vs ≤2/wk)	-0.28 (-0.92 to 0.36)	0.389
	PA: Insufficient (vs Inactive)	-0.33 (-1.40 to 0.73)	0.538
	PA: Meeting guidelines (vs Inactive)	-0.23 (-1.31 to 0.84)	0.670
	Sleep: ≤5 h (vs 6-8 h)	0.52 (-0.54 to 1.59)	0.337
	Sleep: >8 h (vs 6-8 h)	-0.02 (-0.62 to 0.58)	0.949
	Soft drinks: 1-3/wk (vs <1/wk)	0.52 (-0.02 to 1.07)	0.060
	Soft drinks: 4-6/wk (vs <1/wk)	1.04 (0.24 to 1.85)	0.011
Excluding cardiometabolic disease	Soft drinks: ≥1/day (vs <1/wk)	1.53 (0.61 to 2.46)	0.001
	Compulsive eating: 1-3/wk (vs <1/wk)	1.22 (0.67 to 1.78)	<0.001
	Compulsive eating: 4-6/wk (vs <1/wk)	2.08 (1.03 to 3.12)	<0.001
	Compulsive eating: ≥1/day (vs <1/wk)	2.77 (1.13 to 4.41)	<0.001
	Breakfast: 3-4/wk (vs ≤2/wk)	-0.09 (-0.84 to 0.67)	0.822
	Breakfast: 5-6/wk (vs ≤2/wk)	-0.19 (-0.92 to 0.54)	0.606
	Breakfast: Daily (vs ≤2/wk)	-0.45 (-1.10 to 0.19)	0.169
	physical_activity_num	0.00 (-0.40 to 0.41)	0.982
	sleep_duration_num	-0.36 (-0.89 to 0.16)	0.175
Numeric ordinal recoding	f_soft_drinks_num	0.46 (0.21 to 0.72)	<0.001
	compulsive_eating_num	1.15 (0.82 to 1.49)	<0.001
	breakfast_wk	-0.06 (-0.17 to 0.05)	0.289

Table S5B. Sensitivity analyses for overweight/obesity (main exposures).

Sensitivity	Term	Estimate (95% CI)	P
	PA: Insufficient (vs Inactive)	0.94 (0.73 to 1.20)	0.597
	PA: Meeting guidelines (vs Inactive)	0.94 (0.73 to 1.21)	0.612
Main model	Sleep: ≤5 h (vs 6-8 h)	1.34 (1.05 to 1.69)	0.016
	Sleep: >8 h (vs 6-8 h)	1.03 (0.81 to 1.31)	0.811
	Soft drinks: 1-3/wk (vs <1/wk)	1.27 (1.04 to 1.55)	0.017

Sensitivity	Term	Estimate (95% CI)	P
	Soft drinks: 4-6/wk (vs <1/wk)	1.27 (1.01 to 1.60)	0.044
	Soft drinks: ≥1/day (vs <1/wk)	1.42 (1.11 to 1.82)	0.005
	Compulsive eating: 1-3/wk (vs <1/wk)	1.24 (1.03 to 1.48)	0.020
	Compulsive eating: 4-6/wk (vs <1/wk)	1.73 (1.36 to 2.19)	<0.001
	Compulsive eating: ≥1/day (vs <1/wk)	1.86 (1.35 to 2.57)	<0.001
	Breakfast: 3-4/wk (vs ≤2/wk)	0.90 (0.73 to 1.11)	0.313
	Breakfast: 5-6/wk (vs ≤2/wk)	0.89 (0.71 to 1.11)	0.297
	Breakfast: Daily (vs ≤2/wk)	0.75 (0.61 to 0.92)	0.007
	PA: Insufficient (vs Inactive)	0.94 (0.73 to 1.20)	0.605
	PA: Meeting guidelines (vs Inactive)	0.91 (0.70 to 1.18)	0.471
	Sleep: ≤5 h (vs 6-8 h)	1.27 (0.98 to 1.64)	0.071
	Sleep: >8 h (vs 6-8 h)	1.06 (0.83 to 1.36)	0.620
	Soft drinks: 1-3/wk (vs <1/wk)	1.27 (1.03 to 1.57)	0.024
	Soft drinks: 4-6/wk (vs <1/wk)	1.28 (1.00 to 1.64)	0.048
Excluding cardiometabolic disease	Soft drinks: ≥1/day (vs <1/wk)	1.50 (1.16 to 1.94)	0.002
	Compulsive eating: 1-3/wk (vs <1/wk)	1.26 (1.04 to 1.51)	0.016
	Compulsive eating: 4-6/wk (vs <1/wk)	1.56 (1.19 to 2.04)	0.001
	Compulsive eating: ≥1/day (vs <1/wk)	1.89 (1.36 to 2.63)	<0.001
	Breakfast: 3-4/wk (vs ≤2/wk)	0.86 (0.69 to 1.08)	0.205
	Breakfast: 5-6/wk (vs ≤2/wk)	0.87 (0.69 to 1.10)	0.251
	Breakfast: Daily (vs ≤2/wk)	0.69 (0.55 to 0.86)	<0.001

Table S6. Education × exposure interaction tests

Exposure	Δdf	F	P-interaction
Physical activity	3	1.70	0.165
Sleep duration	3	3.02	0.029
Soft drinks	4	1.00	0.406
Compulsive eating	4	1.46	0.212
Breakfast frequency	4	1.40	0.232

Table S7. Education-stratified sensitivity analysis

Table S7A. Descriptive BMI and overweight/obesity prevalence by education level.

Stratum	n	BMI (mean ± SD)	OW/ob prevalence
Non-university	266	24.4 ± 4.7	39.5%
University	936	23.7 ± 4.1	30.4%

Table S7B. Education-stratified fully adjusted associations of soft-drink intake and compulsive eating with BMI (Model 2). Same covariates as main model; sparse regions collapsed within strata.

Stratum	Term	β (95% CI)	P
Non-university (n = 266)	Soft drinks: 1-3/wk (vs <1/wk)	0.14 (-1.29 to 1.57)	0.846
Non-university (n = 266)	Soft drinks: 4-6/wk (vs <1/wk)	1.30 (-0.90 to 3.51)	0.246
Non-university (n = 266)	Soft drinks: ≥1/day (vs <1/wk)	1.93 (0.01 to 3.84)	0.048
Non-university (n = 266)	Compulsive eating: 1-3/wk (vs <1/wk)	1.30 (-0.26 to 2.87)	0.102
Non-university (n = 266)	Compulsive eating: 4-6/wk (vs <1/wk)	2.44 (0.26 to 4.61)	0.028
Non-university (n = 266)	Compulsive eating: ≥1/day (vs <1/wk)	1.53 (-2.42 to 5.49)	0.446
University (n = 936)	Soft drinks: 1-3/wk (vs <1/wk)	0.72 (0.12 to 1.32)	0.020
University (n = 936)	Soft drinks: 4-6/wk (vs <1/wk)	1.03 (0.17 to 1.89)	0.019
University (n = 936)	Soft drinks: ≥1/day (vs <1/wk)	1.14 (0.08 to 2.20)	0.035
University (n = 936)	Compulsive eating: 1-3/wk (vs <1/wk)	1.20 (0.62 to 1.77)	<0.001
University (n = 936)	Compulsive eating: 4-6/wk (vs <1/wk)	2.71 (1.54 to 3.87)	<0.001
University (n = 936)	Compulsive eating: ≥1/day (vs <1/wk)	3.17 (1.27 to 5.08)	0.001
